# Localization of the Cluster satellites in the geospace environment

**DOI:** 10.1038/s41597-025-04639-z

**Published:** 2025-02-22

**Authors:** Benjamin Grison, Fabien Darrouzet, Romain Maggiolo, Mychajlo Hajoš, Michal Dvořák, Martin Švanda, Anna Jeřábková, Matthew Graham George Thaddeus Taylor, Delphine Herment, Arnaud Masson, Jan Souček, Ondřej Santolík, Johan De Keyser

**Affiliations:** 1 Institute of Atmospheric Physics of the Czech Academy of Sciences (IAP), Department of Space Physics, 14100 Prague, Czech Republic; 2 Royal Belgian Institute for Space Aeronomy (BIRA-IASB), Department of Space Physics, Brussels, Belgium; 3 European Space Research and Technology Centre (ESTEC), Noordwijk, The Netherlands; 4 European Space Astronomy Centre (ESAC), Madrid, Spain

**Keywords:** Magnetospheric physics, Magnetospheric physics, Solar physics

## Abstract

The geometry of the terrestrial magnetized environment, or geospace, varies widely in space and time due to the Earth’s magnetic field interactions with the interplanetary medium. A spacecraft’s location in geospace is only approximately determined by its coordinates since the environment is inhomogeneous, with distinct physical processes occurring in different regions. Knowing the location in the geospace offers a strong support for data analysis. This paper introduces a new dataset, Geospace Region and Magnetospheric Boundary identification (GRMB), which provides labelled positions for each Cluster spacecraft over the whole mission, with respect to the local environment. This continuous labelling is based on manual selection, supported by browsing 44 different Cluster data products. The GRMB dataset includes 15 labels spanning from the plasmasphere to solar wind regions. Its consistency is validated over 7 years against reference lists and by the physical properties of the GRMB regions. Over those years, Cluster spent a similar proportion of the time (≈15%) in the regions labelled lobe, plasmasheet, plasmasheet transition region, magnetosheath and solar wind.

## Background & Summary

The proper classification of plasma regions and boundaries crossed by spacecraft of any magnetospheric mission is necessary to perform unambiguous statistical studies of fundamental plasma processes occurring in a particular region, such as refilling in the plasmasphere, reconnection at the magnetopause, turbulence in the magnetosheath or wave propagation in the magnetosphere. The magnetosphere environment is highly dynamic and its regions cannot be identified by the orbital information alone. This paper describes a comprehensive dataset, providing the location of all 4 Cluster satellites^1–3^ during the whole duration of the mission, not as coordinates, but by labeling the surrounding environment. This results in a dataset called Geospace Region and Magnetospheric Boundary identification (GRMB).

Recently, several automated identification methods have been developed to classify regions crossed by satellites. Bakrania, *et al*.^4^ used a neural network model to classify different electron populations within a reduced Cluster dataset into plasmasheet, plasmasheet boundary layer and lobe categories. Automatic classification methods have been developed to identify 10 near-Earth regions crossed by the 4 MMS (Magnetospheric MultiScale) satellites^5^ or to identify three regions (magnetosphere, magnetosheath and solar wind) from four different spacecraft missions^6^. 8 years of MMS dayside observations have been classified into 4 plasma regions (magnetosphere, magnetosheath, solar wind, and ion foreshock) with an unsupervised machine learning tool^7^. These methods usually do not provide a classification when a single parameter is missing.

On the contrary, the GRMB dataset is built with a non-automatic method, in order to combine different datasets over time and to use the human capabilities to interpret the huge amount of information provided by the Cluster instruments, in particular the time-frequency and time-energy spectrograms. The Cluster mission^1^ consists of 4 identical spacecraft that were launched in 2000 into near polar inclined elliptical orbits, with a period of 57 hours, an initial perigee of  ~ 4.0 *R*_*E*_ and an initial apogee of  ~ 19.6 *R*_*E*_. All 4 spacecraft have been in operation until September 2024. During the course of this very long mission all 11 instruments have data gaps and some of them have failed: the GRMB classification relies then on other instruments. The GRMB dataset aims at providing the largest possible coverage of the Cluster location by limiting the non-classified intervals to the periods when no data products are available.

Our manual selection of the the regions and boundaries offers a powerful tool to the scientific community to analyze the Cluster dataset. An immediate interest of the GRMB dataset is to provide a continuous coverage of the spacecraft location when browsing data. The scientific utility of this dataset has already been demonstrated in the frame of a magnetopause study^8^. These are the reasons why the European Space Agency (ESA) made the dataset publicly available on the Cluster Science Archive (CSA, https://csa.esac.esa.int/csa-web/#search)^9^.

The GRMB dataset contains 15 different labels, 4 labels covering the regions outside the magnetosphere, 9 regions inside the magnetosphere, and 2 for no-data intervals and unknown properties. The dataset has been built based on a visual inspection of pre-generated plots of 44 Cluster products coming from 6 different instruments and auxiliary data.

The labels (see Table 1) define regions (IN/PLS, IN/PSH, IN/LOB, OUT/MSH and OUT/SWF), transition regions (IN/PPTR, IN/PSTR, IN/POL, IN/MP, IN/MPTR, IN/BSTR) and puzzling regions (IN/UKN, OUT/UKN, UNKNOWN, N/A). The transition regions are overlapping with the regions in order to include the entire boundaries. It means that the regions are more homogeneous than the transition regions.

**Table 1:** List of the 15 GRMB items that describe the regions and transition regions crossed by the Cluster satellites during the mission.

Index	Label	Name	Description
1	IN/UKN	INside the magnetosphere	Region located for sure inside the magnetosphere with no evident properties from any other IN label
2	IN/PLS	PLasmaSphere	Region of dense plasma close to the perigee
3	IN/PPTR	PlasmaPause TR	Region of strong plasma density gradient close to the perigee
4	IN/PSH	PlasmaSHeet	Region with isotropic fluxes of energetic particles inside the magnetosphere
5	IN/PSTR	PlasmaSheet TR	Transition region in between the plasmasheet and the lobes
6	IN/LOB	LOBe	“Empty” regions (low particle fluxes, no wave activity) inside the magnetosphere
7	IN/POL	POLar regions	Magnetosheath-originating plasma and a dipolar magnetic field
8	IN/MP	MagnetoPause	Sharp transition/boundary between the magnetosheath and the magnetosphere
9	IN/MPTR	MagnetoPause TR	Long and complex transition region between the magnetosheath and the magnetosphere (typically more than one crossing or superposition of magnetosheath and magnetosphere properties)
10	OUT/MSH	MagnetoSHeath	Intense wave activity, no dipolar magnetic field. Higher n and B and lower v than in the closest SWF
11	OUT/BSTR	Bow Shock TR	Transition region where the properties are switching from MSH to SWF
12	OUT/SWF	Solar Wind and Foreshock	Lower n and B and higher v than in the closest MSH
13	OUT/UKN	OUTside the magnetosphere	Region located for sure outside the magnetosphere with no evident properties from any other OUT label
14	UNKNOWN	Unknown	Time interval when the available data cannot be conclusively interpreted
15	N/A	Void	Time interval with no available data (in the pre-generated CSA plots)

TR, n, B, and v stand for transition region, density, magnetic field, and velocity, respectively.

The comparisons with reference event lists, automatic classification and predicted positions show that labels correspond to the expected regions they are designating. IN/PLS, IN/PPTR, IN/MPTR, IN/MP, OUT/MSH OUT/BSTR, OUT/SWF are usually straightforward to classify. IN/PSTR, IN/PSH, IN/LOB and IN/POL are the regions that are the most difficult to properly identify. If few data are available it might be IN/UKN, OUT/UKN or less frequently UNKNOWN. N/A is used only when none of the products was available at that time. The time resolution for the regions is approximately 20 minutes: short region changes are not resolved. Sharp transition regions can be shorter. However, IN/MPTR and IN/PSTR can last several hours.

The GRMB dataset intends to cover the full Cluster mission duration (January 2001 to September 2024). Years 2001-2022 have already been delivered to the CSA. Years 2023 and 2024 shall be processed in 2025 and 2026, respectively. This paper serves as a User Guide to the Cluster GRMB dataset.

## Methods

### Methodology

**Fig. 1:**
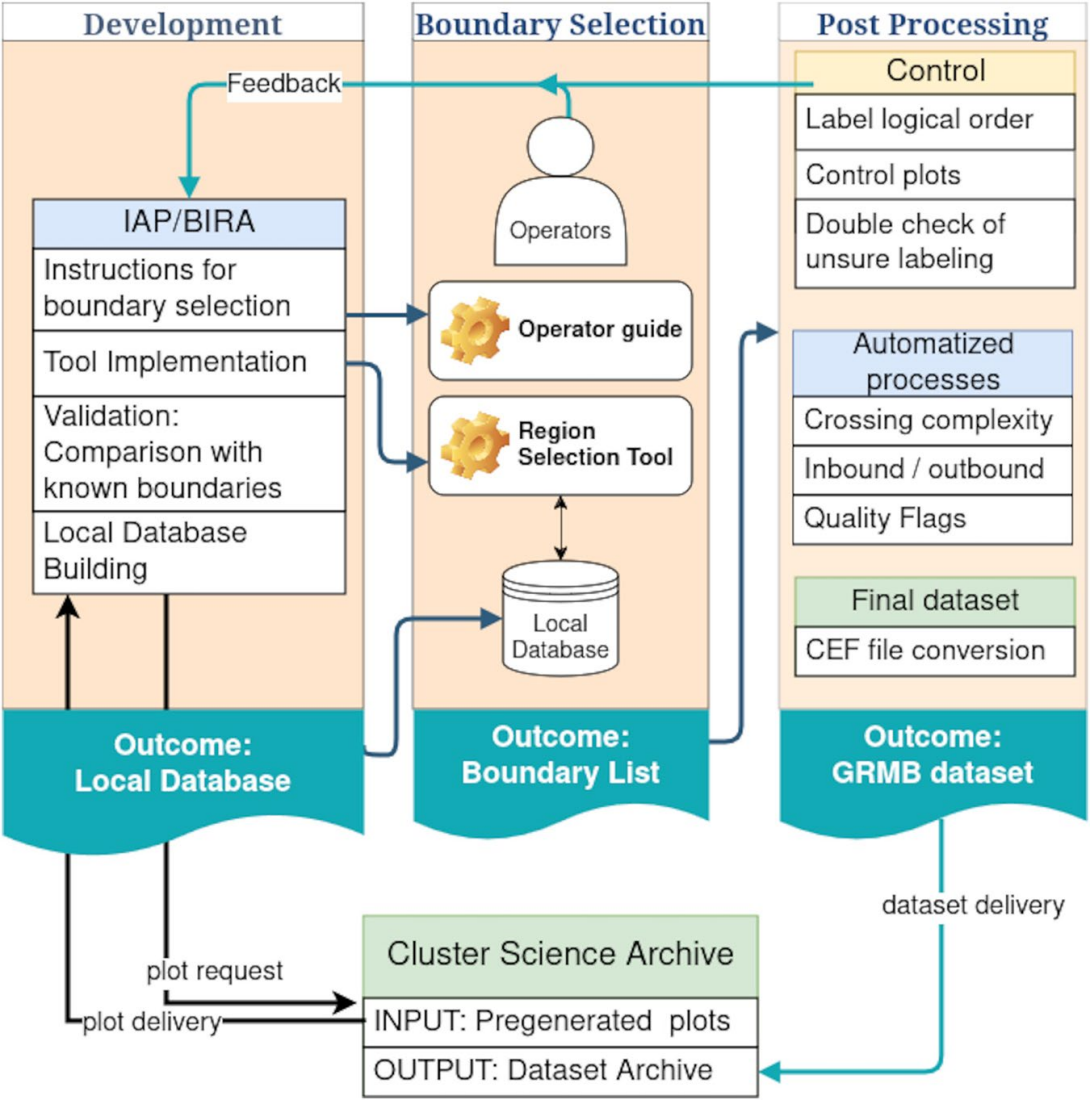
Methodology of the project. Input and output are CSA products. The tasks can be split into three categories: the Region Selection Tool development (left), the Boundary selection (middle) and the post-selection processing (right).

The methodology is summarized in Fig. 1. Inputs of this project are pre-generated plots^10^ produced at the CSA (https://csa.esac.esa.int/csa-web/#graph). These plots are generated at three time-resolutions (1 day, 6 hours and 1 hour) for more than 40 instrument datasets.

The first task of this project was to build a local database gathering all the plots from the 44 products listed in Table 2 from auxiliary products and 6 instruments: PEACE^11^, CIS^12^, EFW^13^, FGM^14^, STAFF^15^, WHISPER^16^.

**Table 2:** Detail of the 44 available CSA products.

Code	Short Name	Instrument	CSA name
1	ILAT	AUX	CG_AUX_PMP_INVAR_LAT_CAA
2	LSHELL	AUX	CG_AUX_PMP_LSHELL_CAA
3	MLT	AUX	CG_AUX_PMP_MLT_CAA
4	DIST	AUX	CG_AUX_SP_DIST_CAA
10	V_hia	HIA	CG_CIS-HIA_ONBOARD_MOMENTS_VGSE_IONS_TOTAL_CAA
11	Vx_hia	HIA	CG_CIS-HIA_ONBOARD_MOMENTS_VGSE_IONS_X_CAA
12	Vy_hia	HIA	CG_CIS-HIA_ONBOARD_MOMENTS_VGSE_IONS_Y_CAA
13	Vz_hia	HIA	CG_CIS-HIA_ONBOARD_MOMENTS_VGSE_IONS_Z_CAA
14	Vphi_hia	HIA	CG_CIS-HIA_ONBOARD_MOMENTS_VGSE_IONS_PHI_CAA
15	Vtheta_hia	HIA	CG_CIS-HIA_ONBOARD_MOMENTS_VGSE_IONS_THETA_CAA
16	N_hia	HIA	CG_CIS-HIA_ONBOARD_MOMENTS_NI_IONS_CAA
17	Els_hia	HIA	CG_CIS-HIA_LS_IONS_PEF_SPEC_CAA
18	Ehs_hia	HIA	CG_CIS-HIA_HS_IONS_PEF_SPEC_CAA
19	PAhigh_hia	HIA	CG_CIS-HIA_PAD_HS_IONS_GE_1000_SPEC_CAA
20	PAlow_hia	HIA	CG_CIS-HIA_PAD_HS_IONS_LE_1000_SPEC_CAA
31	POT	EFW	CG_EFW_L3_P_CAA
41	Btot	FGM	CG_FGM_BMAG_CAA
42	Bx	FGM	CG_FGM_BGSE_X_CAA
43	By	FGM	CG_FGM_BGSE_Y_CAA
44	Bz	FGM	CG_FGM_BGSE_Z_CAA
51	N_peace	PEACE	CG_PEA_MOMENTS_NE_CAA
52	V_peace	PEACE	CG_PEA_MOMENTS_VGSE_TOTAL_CAA
53	Vx_peace	PEACE	CG_PEA_MOMENTS_VGSE_X_CAA
54	Vy_peace	PEACE	CG_PEA_MOMENTS_VGSE_Y_CAA
55	Vz_peace	PEACE	CG_PEA_MOMENTS_VGSE_Z_CAA
57	Eall_peace	PEACE	CG_PEA_PITCH_SPIN_OMNI_POT_CAA
58	PAmid_peace	PEACE	CG_PEA_PAD_SPEC_CAA
59	PAlow_peace	PEACE	CG_PEA_PAD_SPEC_LOW_CAA
60	PAhigh_peace	PEACE	CG_PEA_PAD_SPEC_HIGH_CAA
71	B2_staff	STAFF	CG_STA_PSD_SPEC_B_MAG_CAA
72	B2_theta	STAFF	CG_STA_PPP_SPEC_THETA_CAA
75	N_whisper	WHISPER	CG_WHI_NE_CAA
76	E2_whisper	WHISPER	CG_WHI_NATURAL_SPEC_FULL_CAA
77	E2a_whisper	WHISPER	CG_WHI_ACTIVE_SPEC_FULL_CAA
80	V_codif	CODIF	CG_CIS-CODIF_MOMENTS_VGSE_H1_TOTAL_CAA
81	Vx_codif	CODIF	CG_CIS-CODIF_MOMENTS_VGSE_H1_X_CAA
82	Vy_codif	CODIF	CG_CIS-CODIF_MOMENTS_VGSE_H1_Y_CAA
83	Vz_codif	CODIF	CG_CIS-CODIF_MOMENTS_VGSE_H1_Z_CAA
84	Vphi_codif	CODIF	CG_CIS-CODIF_MOMENTS_VGSE_H1_PHI_CAA
85	Vtheta_codif	CODIF	CG_CIS-CODIF_MOMENTS_VGSE_H1_THETA_CAA
86	N_codif	CODIF	CG_CIS-CODIF_MOMENTS_NI_H1_CAA
87	Eh_codif	CODIF	CG_CIS-CODIF_H1_PEF_SPEC_CAA
88	PAhigh_codif	CODIF	CG_CIS-CODIF_PAD_H1_GE_1000_SPEC_CAA
89	PAlow_codif	CODIF	CG_CIS-CODIF_PAD_H1_LE_1000_SPEC_CAA

The second task was to develop a light tool that allows an efficient vignette browsing and a manual region/boundary selection. This tool is not further described here as it has no functionality that impacts label selections. Nevertheless many figures are screenshots taken from the tool user interface (see Figs. 3 to 7). These figures present the three panel combinations, or views, that are mainly used for the selection: *IN-Plasmasphere* view, *IN-Magnetosphere* view and *OUT regions* view.

The boundary selections are made by operators by visually selecting the matching label descriptions given in the next section. When the operator is not confident with his choice he has the possibility to mark the boundary for double-checking by a supervisor before validation. The operator can also mark the boundary as complex: this sets the *crossing_complexity* variable value to 1. We developed two control routines. The first one is an automatic procedure detailed in the *Forbidden consecutive items* subsection that catches the forbidden consecutive pair regions (MSH cannot follow PPTR, for example). The second one are routine plots displaying the selection made for the four spacecraft during two consecutive orbits. However, as for any eye-made selection, one cannot rule out that some mistakes can go through the validation process.

Then, we routinely generate the CEF (Cluster Exchange Format) files by compiling the boundary selection and adding the automatically set fields.

### Description of the GRMB items

In the GRMB project, we have defined 15 labels to describe the regions and boundaries crossed by the Cluster satellites during the entire mission. But, as “boundary” is a widely used term and can lead to some confusion, we decided to use instead the “transition region” (TR) denomination. As there is no assumption about the nature of the boundary, a transition region is larger than the boundary itself and includes part of the nearby regions. This large transition region classification is useful for scientists who plan to study a boundary with their own identification criteria.

The number of 15 items is arbitrary. It is large enough to describe geospace and small enough to allow a fast by-eye selection. The names of those 15 items are listed in Table 1. We refer to those items by their labels in this section, and by their shortened labels later in the other sections (without the IN/ and OUT/ prefix where applicable). The items are found either inside the magnetosphere (9 items starting with IN) or outside the magnetosphere (4 items starting with OUT). Three items can contain the IN/OUT boundary (IN/MP, IN/MPTR, and IN/POL).

The last two items correspond to time periods when the spacecraft location is unknown (UNKNOWN) or when no data is available (N/A). These two items are mandatory for the continuous coverage of the labelling. There are five labels for regions (IN/PLS, IN/PSH, IN/LOB, OUT/MSH, and OUT/SWF), five for transition regions (IN/PPTR, IN/PSTR, IN/MP, IN/MPTR, and OUT/BSTR) and four for undefined regions (IN/UKN, OUT/UKN, UNKNOWN, and N/A). The IN/POL label can be either a region (where well inside the magnetosphere) or a transition region (where close to the IN/OUT boundary).

The 15 GRMB items can be described in the following way.

#### IN/UKN: Inside the magnetosphere

In the magnetosphere, the pressure is dominated by the Earth’s magnetic field pressure, or by the pressure of trapped plasma in the central plasmasheet far enough from the Earth. The magnetosphere contains various sub-regions with different characteristics. However, all of these regions can be distinguished from the magnetosheath and solar wind because they have a strong and stable magnetic field compared to the one in the solar wind and magnetosheath and/or because of the more stable particle pitch-angle distribution inside the magnetosphere than outside (resulting from the frozen magnetic field in the solar wind plasma).

**Fig. 2:**
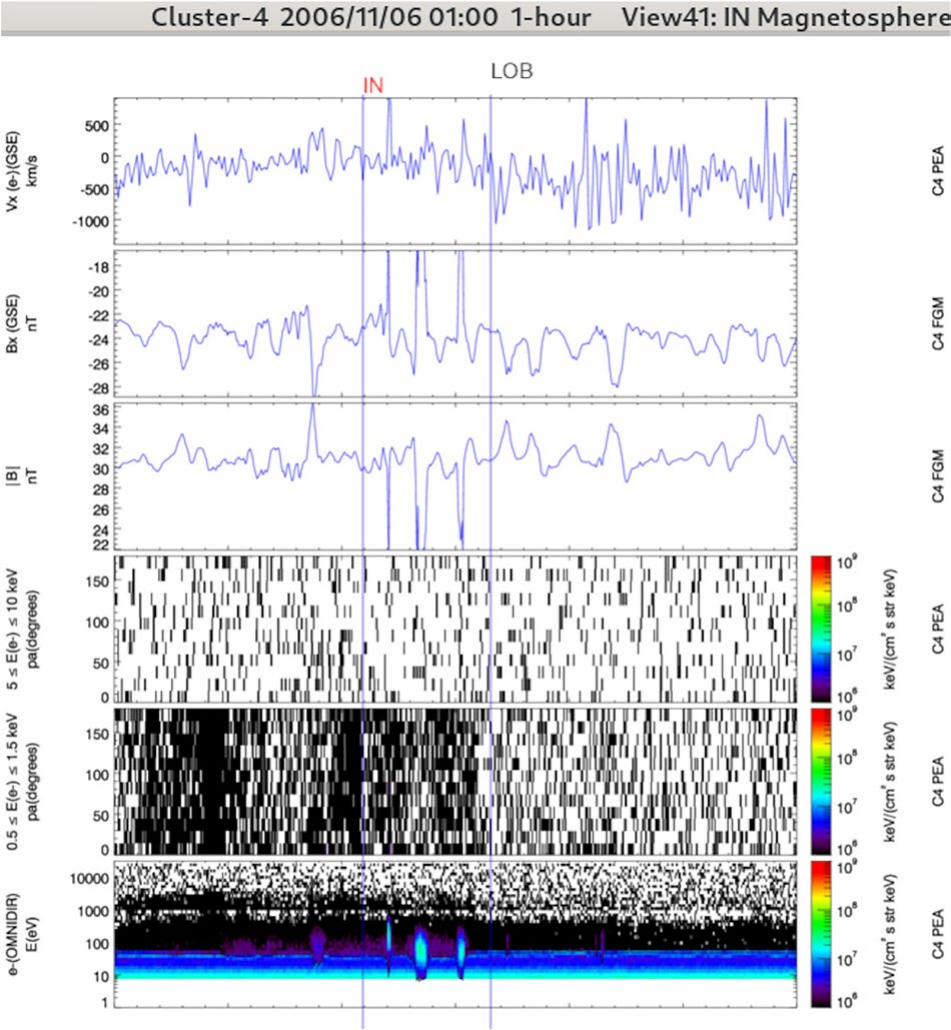
Overview of an inside unknown region (IN/UKN) crossed by Cluster-4 during a 1-hour interval plotted with the Region Selection Tool (RST) developed to visualize the CSA products and to build the GRMB dataset. The top bar displays the spacecraft number, the window start time and duration, and the displayed panel combination (view). The vertical blue lines delimit different records. The record label is seen above the vertical line that marks the start time. Red labels indicate that the operator selected a complex crossings (mixing of region properties in the present case). This panel combination is well-adapted for the IN regions (IN/PSH, IN/PSTR, IN/LOB, and somehow IN/POL). From top to bottom it presents the x-component of the plasma bulk velocity (from PEACE in the present case), the x-component of the magnetic field (FGM), the amplitude of the magnetic field (FGM), the pitch-angle dependence of high-energy particle fluxes (from PEACE in the present case), the pitch-angle dependence of low-energy particle fluxes (from PEACE in the present case) and the electron time-energy spectrogram (PEACE).

The label IN/UKN corresponds to unidentified magnetospheric regions, i.e. to regions located inside the magnetosphere but that do not fulfill the criteria of the other magnetospheric regions discussed hereafter (cf. Fig. 2). It occurs when few data products are available or when properties from different items are mixed up.

IN/UKN can be adjacent to any other magnetospheric region or to the magnetosheath (OUT/MSH).

#### IN/PLS: Plasmasphere

The plasmasphere is the upward extension of the Earth’s ionosphere. It is a torus-like region populated by dense and cool plasma (density between 10 and 10^4^ *cm*^−3^ and temperature of the order of 10^4^ *K*) surrounding the Earth^17^. This region extends out to several Earth radii (*R*_*E*_). The plasmasphere overlaps, or is otherwise in close proximity, with the hot ( ≈ 100 *eV* − 100 *keV*) tenuous ( ≈ 1 *cm*^−3^) plasma of the plasmatrough or the plasma sheet and ring current^18^. The plasmasphere is bounded by the plasmapause.

**Fig. 3:**
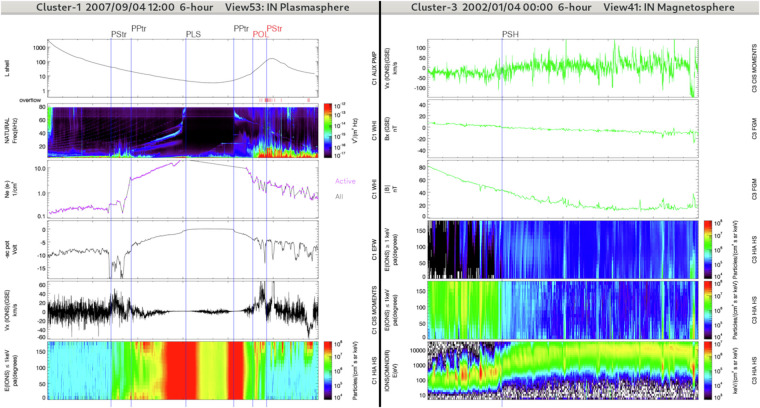
Left: Overview of a plasmasphere (IN/PLS) and 2 plasmapauses (IN/PPTR) crossed by Cluster-1 during a 6-hours interval (see Fig. 2 for details). The panel combination is optimized for the plasmasphere and plasmapause regions. From top to bottom it presents spacecraft L-shell value (auxiliary data), the electric field spectrogram (WHISPER), plasma density measurements (from WHISPER in the present case), the spacecraft potential (EFW), the x-component of the plasma bulk velocity (from CIS/HIA in the present case), and the pitch-angle distribution of low-energy particle fluxes (from CIS/HIA in the present case). The other labels (IN/PSTR and IN/POL) have been selected with the panel combination detailed hereafter. Right: Overview of a plasmasheet (IN/PSH) crossed by Cluster-3 during a 6-hours interval (same format and panel combination as Fig. 2). This panel combination is well-adapted for the IN regions (IN/PSH, IN/PSTR, IN/LOB, and somehow IN/POL).

In the GRMB dataset, the IN/PLS label is selected when the plasma frequency is above the upper frequency limit of the WHISPER instrument (80 *kHz*) and the spacecraft is close to perigee (see Fig. 3). The main characteristic of the plasmasphere is its high density of cold plasma as compared to other regions of the magnetosphere, a density typically higher than 70 *cm*^−3^ measured by the WHISPER instrument. The identification can be confirmed by observations of an isotropic cold plasma population with a non-fluctuating low plasma bulk velocity. In case of region overlap, the priority goes to the IN/PLS label (see Subsection *Regions overlap*). It means that we do not search for any other regions as long as the satellite is in a IN/PLS region: the data can be difficult to interpret inside the plasmasphere because the payload was not designed to probe these regions. For example the CIS ion detectors can be contaminated by radiation belts high energy particles during years with a lower perigee^19^.

IN/PLS regions are usually surrounded by IN/PPTR regions. In case of missing data it can also be IN/UKN or N/A.

#### IN/PPTR: Plasmapause transition region

The plasmapause transition region is the outer boundary layer of the plasmasphere (see Fig. 3, left panels). It has been first defined as a boundary layer by Carpenter and Lemaire^20^: “There may be substantial spatial and temporal variations in the particular plasma populations that are in juxtaposition in the plasmapause region, as well as a substantial variety in the plasmapause density structure itself".

The label IN/PPTR is identified by a plasma density gradient observed close to perigee. The density gradient can be seen either in the particle density measurements, in the plasma frequency, or in the spacecraft potential. Note that there is no fixed threshold in the value of the plasma frequency, or the density, or the potential to determine the IN/PPTR start and end times.

The plasmaspheric plumes as well as any of other density structures located close to the plasmapause are included in this region as long as they display a noticeable density enhancement. The IN/PPTR can be adjacent to the IN/PLS and to any other IN item, with a preference for the plasmasheet transition region (IN/PSTR).

Similarly to IN/PLS, we do not search for any other regions until the spacecraft exits IN/PPTR.

#### IN/PSH: Plasmasheet

The plasmasheet is populated by hot (typically in the *keV* to tens of *keV* range) isotropic plasma originating both from the ionosphere and solar wind and trapped on closed magnetic field lines^21,22^. Compared with the magnetospheric lobes, the plasmasheet has the following characteristics: higher *β*, higher plasma density and higher energy particles (see Fig. 3, right panels). The plasmasheet is not a uniform region but is rather layered with regions of different plasma regimes. Its properties (plasma *β*, temperature and density) vary from the inner to the outer plasmasheet.

In the nightside plasmasheet, the Cluster satellites orbit at lower geocentric distances than the reconnection X-line in the magnetotail, which is typically located at geocentric distances between -20 and -30 *R*_*E*_, even if it can occur between -7 and -10 *R*_*E*_^23^. Thus, in the nightside plasmasheet at the level of Cluster orbit, the plasma convection is mostly directed Earthward, contrary to the magnetosheath where it is mostly antisunward.

The main IN/PSH characteristics are isotropic fluxes of energetic particles inside the magnetosphere and outside the plasmasphere. Our definition of the plasmasheet includes the nightside plasmasheet, the dayside plasmasheet, and also the ring current region. IN/PSH is thus not limited to a sheet. When the particle properties change in the region of B_x_-sign reversal, the IN/PSH label is set to IN/PSH complex rather than to IN/UKN.

#### IN/PSTR: Plasmasheet transition region

**Fig. 4:**
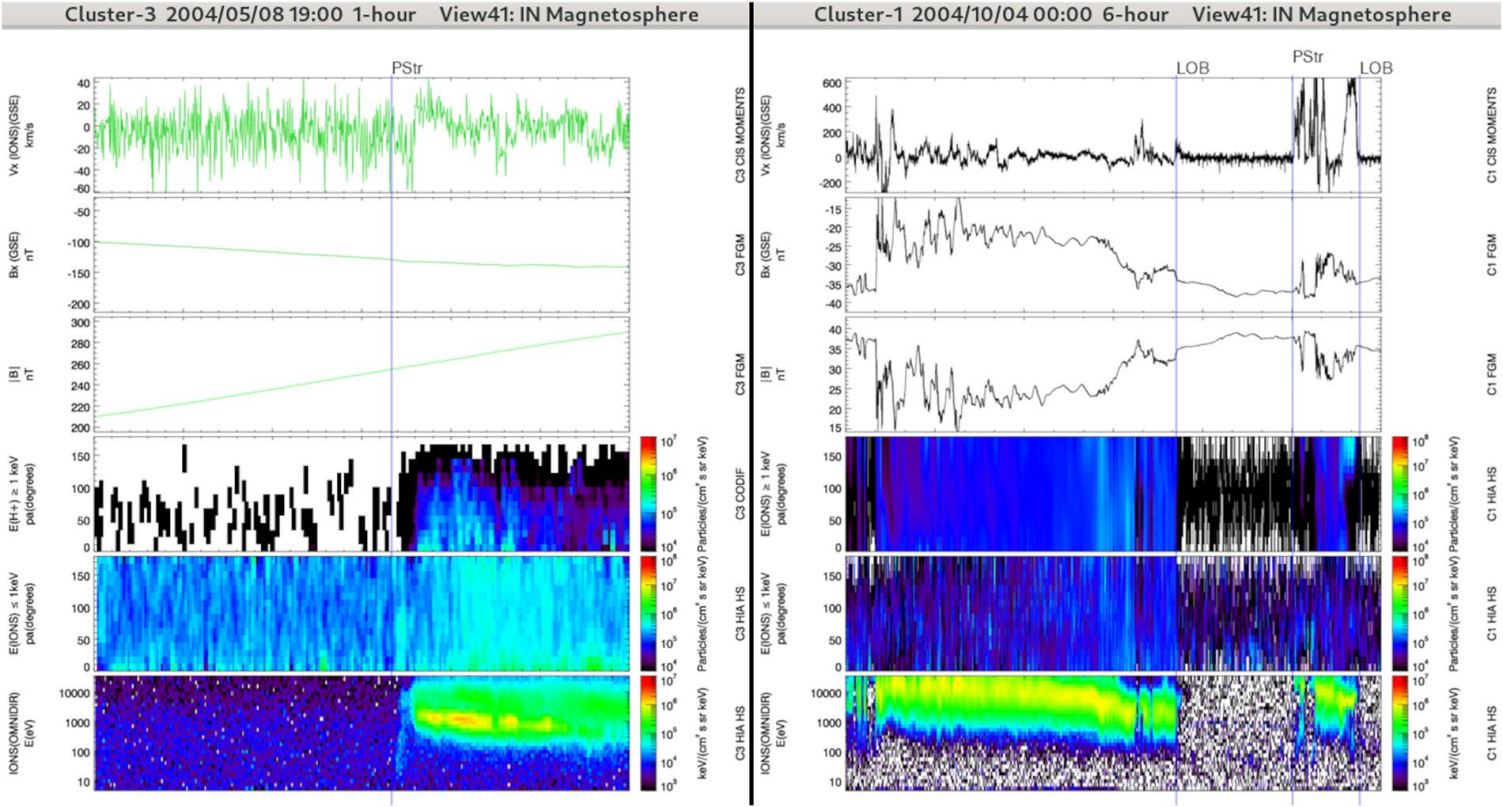
Overview of lobe regions (IN/LOB) and plasmasheet transition regions (IN/PSTR) crossed by Cluster-3 (left panel) and Cluster-1 (right panel) during two distinct 6-hours intervals (same format and panel combination as in Fig. 2). The start regions are IN/PSTR for the right panel and IN/LOB for the left panel.

The plasmasheet transition region refers to the transition region in between the plasmasheet and the lobes (see Fig. 4). It includes the plasmasheet boundary layer that is located on recently reconnected magnetic field lines. The plasmasheet boundary layer, at the interface of the lobes and plasmasheet, is a region characterized by a population of field-aligned particles while the plasmasheet is associated with isotropic plasma. However, in some cases the lobe-plasmasheet interface is characterised by the absence of field-aligned ion beams^24^. There is no consensus on the presence of a boundary layer in this situation, some authors arguing that there is no transition region between the lobes and the plasmasheet^25^ while other authors claim that a transition region distinct from the plasmasheet actually exists even in the absence of ion beams^26^.

The IN/PSTR region may not be limited to the plasmasheet boundary layer itself, it may also include the outer plasmasheet region and some intervals in the lobe region in particular when the Cluster spacecraft are alternatively flowing through the lobes and outer plasmasheet due to the flapping motion of the magnetotail.

In our dataset IN/PSTR displays particle fluxes less isotropic than IN/PSH. When anisotropic ion fluxes are present we consider IN/PSTR when the main fluxes are above 500 *eV*. Note that anisotropic ion fluxes close to the magnetopause, mainly of magnetosheath origin, observed inside the magnetosphere can be labeled as IN/MPTR, IN/POL, or IN/PSTR. It is specially true when the magnetopause itself is not seen during the orbit. The energy level classification is even more complicated when no ion data are available.

Thus the presence of intense ion fluxes above 1 *keV* is used to discriminate the plasmasheet from the magnetosheath together with the ion pitch angle distribution. Isotropic ion fluxes above 1 *keV* are associated with IN/PSH while anisotropic ion fluxes above 1 *keV* are associated with IN/PSTR. In addition, if the ion flux above 1 *keV* varies rapidly we identify the region as IN/PSTR as it may correspond to periods when Cluster skims the outer plasmasheet and -at least partially- enters the lobe region. Note that the plasmasheet temperature may be low and similar to the magnetosheath temperature during periods when the plasmasheet is dominated by *H*^+^ ions of magnetosheath origin that haven’t been significantly energized. This can happen in the far magnetotail or during prolonged periods of northward IMF^27^ which has been attributed to the high influx of solar wind/magnetosheath ions^28,29^. This means that the distinction between IN/PSTR, IN/MPTR and IN/POL is sometimes difficult.

#### IN/LOB: Lobe

The lobe refers to the region of open magnetic field lines at high latitude connecting the ionosphere to the solar wind. It contains low density cold plasma consisting of up-flowing ionospheric ions and electrons (the polar wind) and precipitating solar wind electrons^30^. Such low energy plasma cannot be detected directly with the CIS experiment due to the spacecraft positive charging that prevents the ions from entering the detector.

The pressure in the magnetospheric lobes is dominated by the magnetic pressure and the plasma *β* is low (typically around 0.05). In the Northern lobe, the magnetic field is mostly oriented along the *X*_*GSE*_ direction, while in the Southern lobe region it is mostly oriented along the  − *X*_*GSE*_ direction.

Energetic ions originating from the footprint of the cusp region can also flow in the lobe region. These ions are energetic enough to overcome the spacecraft positive electric potential and be detected by the CIS experiment^31^. Locally accelerated ionospheric ion beams^32^ are also detected in the lobe region, mostly during periods of northward IMF (Interplanetary Magnetic Field). Also during periods of northward IMF, the plasmasheet can protrude in the lobe region, complexifying its geometry^33^.

Lobe plasma is cold and flowing at relatively low velocity. The magnetic field in the lobe region is relatively stable and strong. The lobe region can thus be identified by a very low density from the CIS experiment when ASPOC is not active and lobe ions cannot be detected by CIS (below 0.03 *cm*^−3^)^32^. The electron density can be used to detect the lobe region. Even if lobe electrons can be detected by PEACE, the lobe density is lower than the density of other magnetospheric regions (typically below 0.03 *cm*^−3^)^4^. Finally the magnetic field is used to identify the lobe region, lobe intervals being associated with a strong and stable magnetic field mostly oriented in the  ±*X*_*GSE*_ direction.

The label IN/LOB corresponds to tenuous regions (low unidirectional fluxes of low-energy particles, no wave activity) inside the magnetosphere. IN/LOB item identification highly relies on the availability of particle data. The main criteria for the lobe identification is the non detection of ions by the CIS experiment. Electrons measurements can also be used as an alternative. However, the electron background level in the lobe is difficult to distinguish from actual measurements of low-energy or tenuous electron populations from other magnetospheric regions. This implies that IN/LOB entries can be less frequent in the last years of the mission.

#### IN/POL: Polar regions

**Fig. 5:**
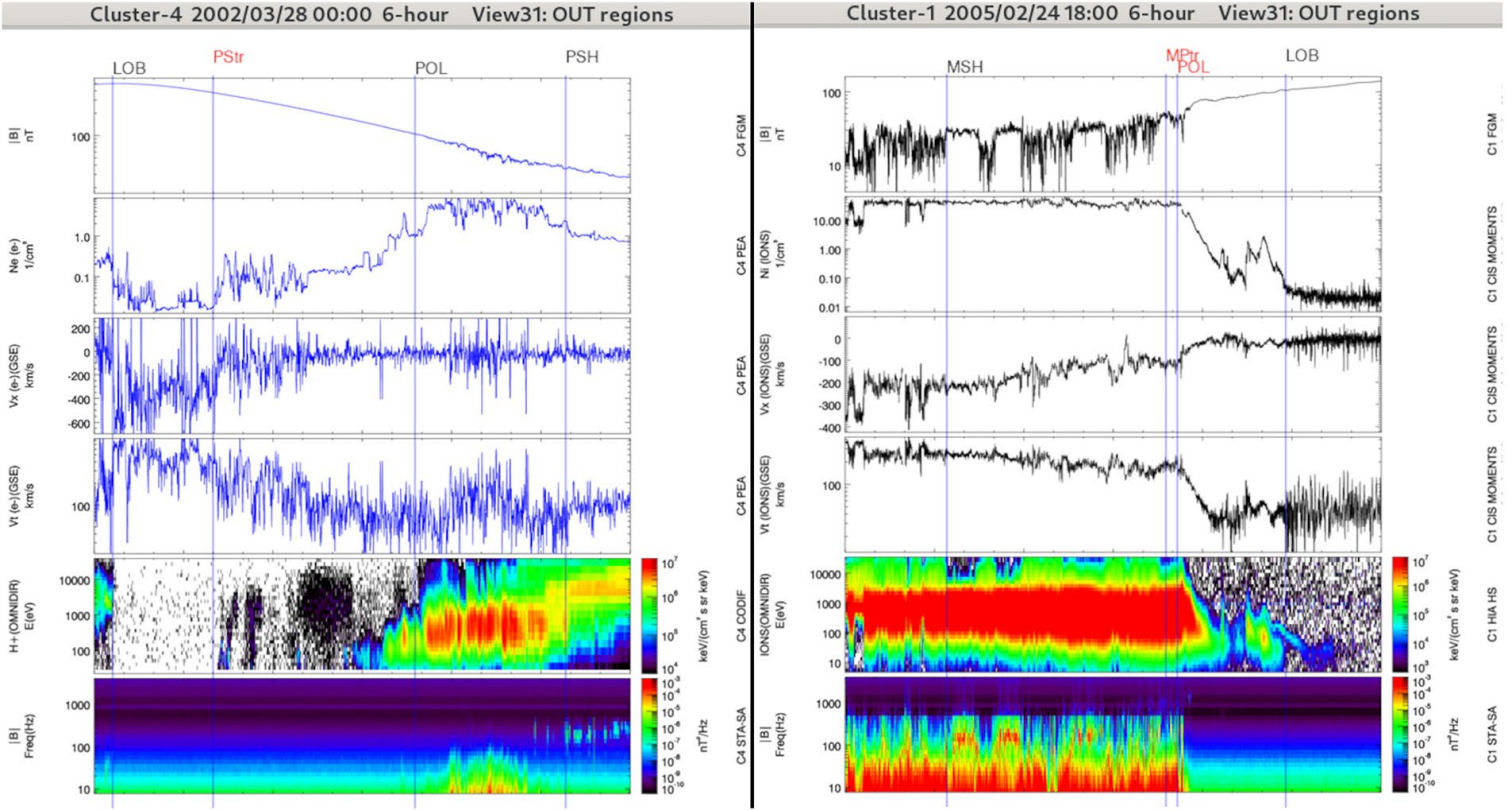
Overview of the polar region (IN/POL) and the neighboring regions crossed by Cluster-4 (left panel) and Cluster-1 (right panel) during two distinct 6-hours intervals plotted with the Region Selection Tool (see Fig. 2 for details). This panel combination is well-adapted for the OUT regions (OUT/MSH, OUT/BSTR, OUT/SWF, as well as for IN/POL). From top to bottom it presents the amplitude of the magnetic field (from FGM), the plasma density (from CIS/HIA in the present case), the x-component of the plasma bulk velocity (from CIS/HIA in the present case), the plasma bulk velocity (from CIS/HIA in the present case), the ion time-energy spectrogram (from CIS/HIA) and magnetic wave power spectral density (from STAFF).

The magnetospheric polar regions contain magnetosheath-like plasma coming along with energy dispersion and observed inside the magnetosphere (see Fig. 5). The main target of the POL item is the polar cusp region which is observed by Cluster close to the magnetopause (distant cusp region) or deep inside the magnetosphere (mid-altitude cusp region)^34^. Based on previous Cluster observations we expect to find the polar cusps within [8-16] MLT^35^.

An IN/POL entry is thus expected to contain magnetosheath-like plasma around 12 MLT (8 to 16 MLT) inside the magnetosphere. An ideal IN/POL entry will display the following characteristics: strong anisotropic ion flux with a typical energy dispersion, and some ULF wave activity in a quasi-dipolar magnetic field region. POL can have a direct boundary with OUT/MSH (Inside-Outside properties).

In the distant regions, one of its boundary will be IN/MP, IN/MPTR or OUT/MSH and the other one will probably be IN/LOB, IN/PSH, IN/PSTR or IN/PPTR. Usually it is observed along the Cluster orbit between January and May. At mid-altitude, IN/POL is not found next to IN/MP, IN/MPTR, nor OUT/MSH. Usually it is observed along the Cluster orbit between July and November. With this definition in MLT, the polar mantle and ion outflows from the polar regions can be found with IN/POL labels when it is next to the cusp, but with a IN/LOB, IN/PSTR or IN/UKN labels when the cusp itself is not seen.

Note that the IN/POL identification is more difficult when ion observations are not available.

#### IN/MP: Magnetopause

The magnetopause refers to the boundary between the magnetosheath and the magnetosphere. It consists of a current sheet associated with sharp variations of the plasma density, velocity and temperature^36^ and in some occasions with a sharp variation of the magnetic field components and magnitude.

Haaland, *et al*.^37^ identified magnetopause crossings by the Cluster spacecraft from a visual identification of abrupt changes in the magnetic or plasma parameters, which are indicative of a transition between the fairly rigid magnetic field and low-plasma density inside the magnetosphere to a more turbulent magnetic field and higher-plasma density in the magnetosheath.

The label IN/MP covers the sharp magnetopause crossings and no other regions are located inside the magnetopause as it corresponds to a boundary, not a transition region. The sharp crossings match a clear rotation of the magnetic field and a change in the plasma population. However, many of the magnetopause crossings are not so obvious and are classified as a magnetopause transition region (next item).

#### IN/MPTR: Magnetopause transition region

**Fig. 6:**
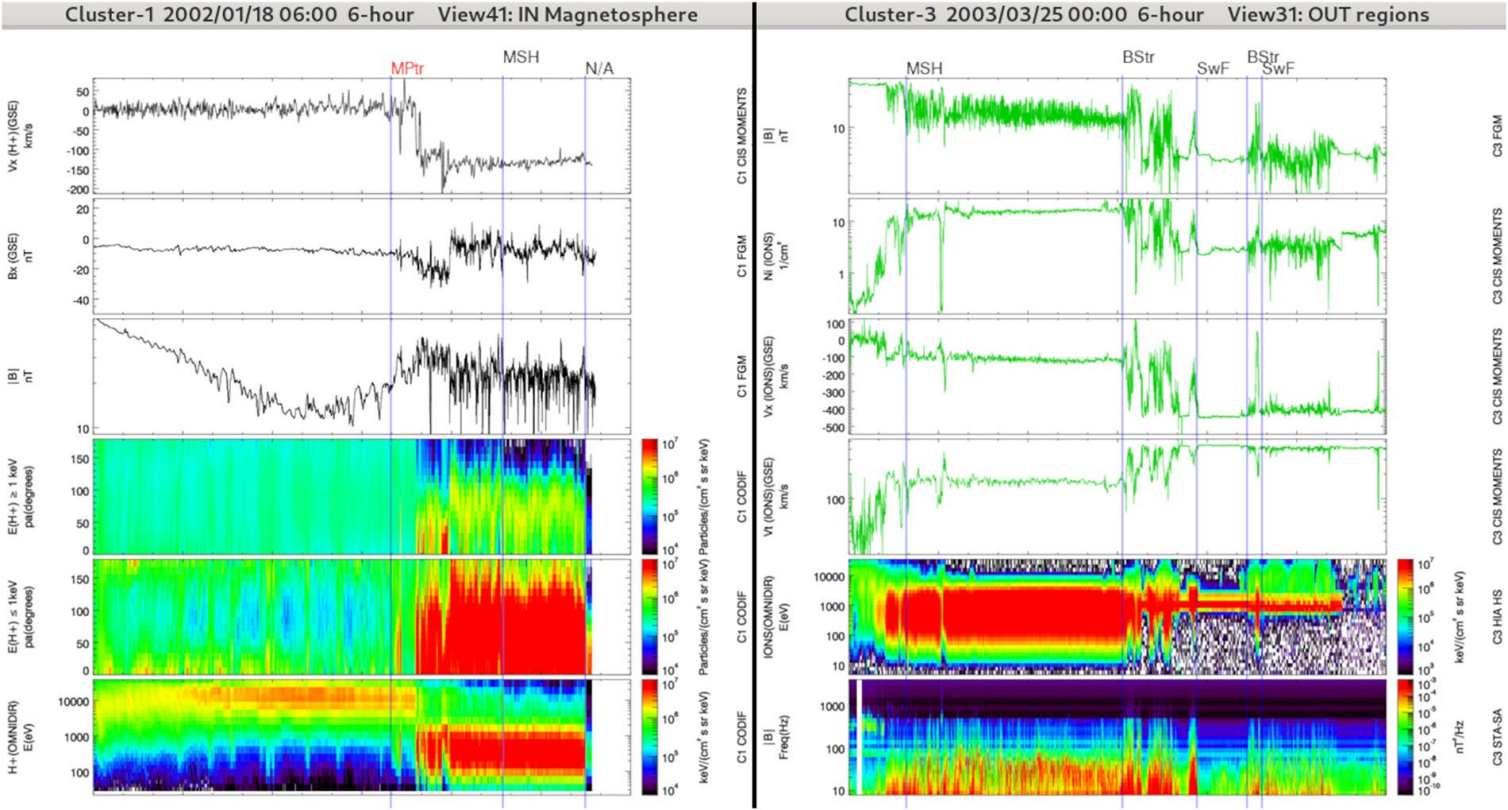
Left: Overview of the magnetopause transition region (MPTR) crossed by Cluster-1 during a 6-hours interval (same format and panel combination as in Fig. 2). Right: Overview of the OUT regions: the magnetosheath (OUT/MSH), the bow shock transition region (OUT/BSTR) and the solar wind and foreshock regions (OUT/SWF) crossed by Cluster-3 during a 6-hours interval. The panel combination is the same as in Fig. 5.

The magnetopause transition region refers to the transition region between the magnetosheath and the magnetosphere, which contains a mixture of plasmasheet and magnetosheath plasma (see Fig. 6, left panels). Due to the motion of the magnetopause and to the Cluster orbit, which may skim the magnetopause, the Cluster satellites may cross repeatedly and sometimes partially the magnetopause making the identification of this boundary complex. In that case, the period associated with such crossings will be classified as IN/MPTR.

The label IN/MPTR includes all the boundary layers adjacent to the magnetopause, excepted the polar cusps (see IN/POL item). The IN/MPTR thus includes the Low and High Latitude Boundary Layer (LLBL and HLBL).

#### OUT/MSH: Magnetosheath

The magnetosheath is the region found between the Earth’s bow shock and the magnetopause^38^. This is a relatively dense region (>10 *cm*^−3^) with a high-level of magnetic fluctuations (see Fig. 6, right panels).

The magnetosheath displays highly variable properties partly depending on the upstream shock orientation (quasi parallel or quasi perpendicular) or on the distance from the Earth-Sun axis.

The OUT/MSH item identification relies mainly on comparisons with the neighboring regions that are always the OUT/SWF (separated with a OUT/BSTR region) and/or the magnetosphere (IN/MP, IN/MPTR or IN/POL).

In OUT/MSH, magnetic field and density values should be higher, and the velocity should be lower, than in the next or previous OUT/SWF region. The wave activity in OUT/MSH is usually higher than in the OUT/SWF and than inside the magnetosphere. In the magnetosphere, the density is lower than in OUT/MSH and the magnetic field evolution is dipolar.

#### OUT/BSTR: Bow shock transition region

The bow shock is the boundary between the solar wind region and the magnetosheath region (see Fig. 6, right panels). The bow shock boundary reacts quickly to the variations of the solar wind dynamic pressure^39^. The bow shock formation and structures are very dynamic^40^. In another study, the bow shock is identified by an “abrupt jump” in the magnetic field (DC and low-frequency waves) and in ion moments (velocity and density)^41^.

The label OUT/BSTR corresponds to a region where the properties are switching from OUT/MSH to OUT/SWF (or vice-versa). Its identification is usually straightforward and it can be very short (single sharp crossing) or last up to a few hours when the (moving) shock is observed multiple times in succession.

#### OUT/SWF: Solar wind and foreshock

The solar wind is continuously blown away by the Sun and surrounds the magnetosheath region. At Earth, it has an average velocity of 500 *km*/*s*. Low-speed solar wind is observed below 400 *km*/*s* and high-speed solar stream above 600 *km*/*s*. The average plasma density is about 5 *cm*^−3^ and the average electron temperature (*T*_*e*_ = 1.4 10^5^ *K*) and ion temperature (*T*_*i*_ = 1.2 10^5^ *K*) are similar^42^.

The foreshock is a region that displays larger fluctuations in the magnetic field, the plasma velocity and the plasma density than in the unperturbed solar wind (see Fig. 6, right panels).

The OUT/SWF item identification relies mainly on comparisons with the neighboring regions that is always a OUT/MSH (separated with a OUT/BSTR region). Magnetic field and density values should be lower than in the next or previous OUT/MSH region. The velocity should be higher than in the nearby OUT/MSH region. The wave activity in OUT/SWF is usually low, a bit more intense in the foreshock than in the solar wind.

#### OUT/UKN: Outside the magnetosphere

**Fig. 7:**
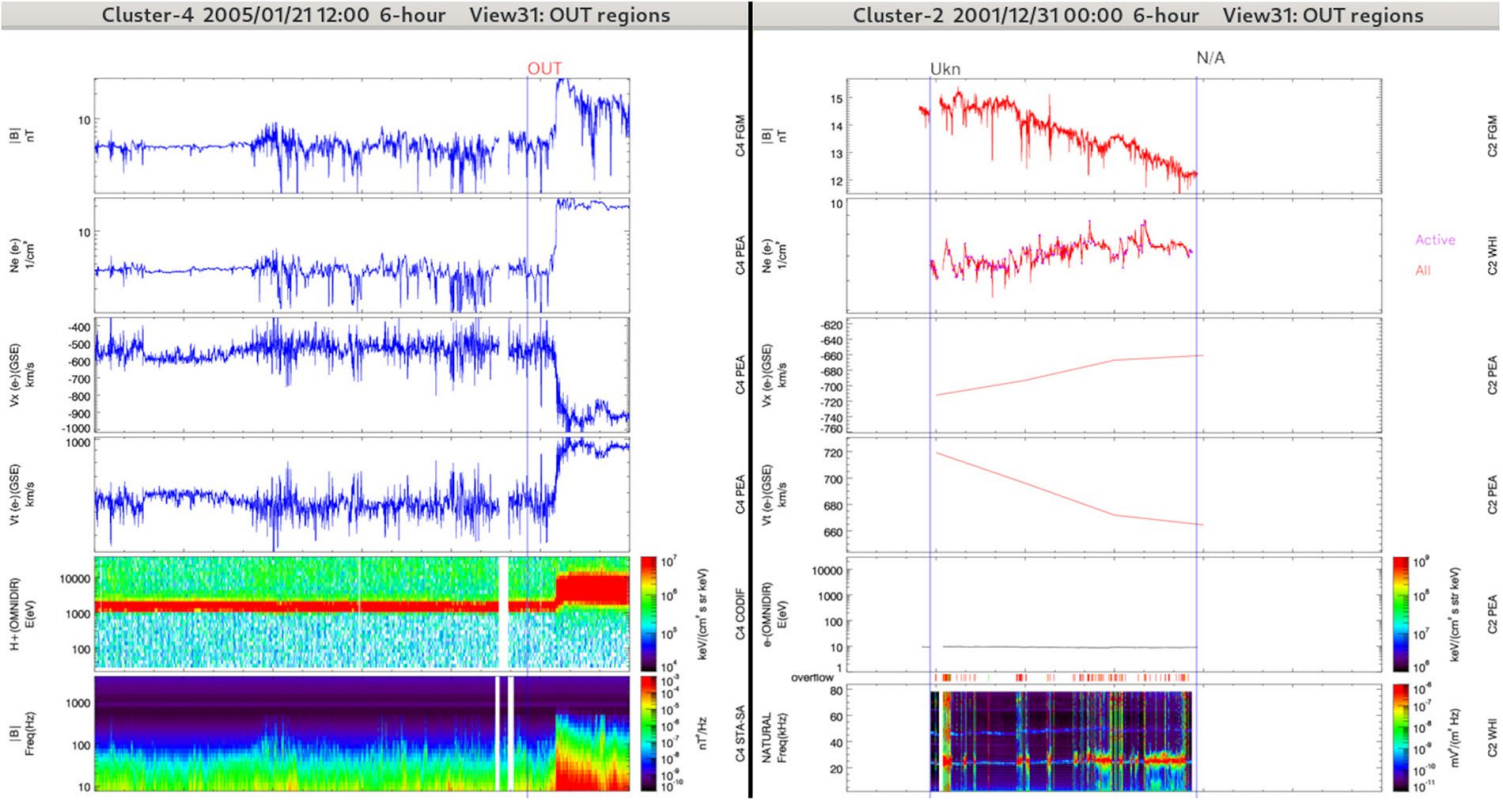
Left panels: Overview of an outside unknown region (OUT/UKN) crossed by Cluster-4 during a 6-hours interval (see Fig. 2 for details). The spacecraft is in the solar wind. The propertie changes at the end of the time window are not the ones expected for a bow shock. The label OUT/UKN (unknown region outside the magnetosphere) is selected. Right panels: Overview of an unknown region (UNKNOWN) crossed by Cluster-2 during a 6-hours interval. The periods with no data available are labeled N/A. Based on the available observations there is an uncertainty in the location of the spacecraft. The label UNKNOWN is thus selected. The panel combinations are the same as in Fig. 5.

The label OUT/UKN corresponds to unidentified regions that are located outside the magnetosphere, i.e. regions that do not fulfill the criteria of the other outer regions discussed above. For example the observations can match with the arrival of an interplanetary coronal mass ejection and there is no such label in the dataset (left panels in Fig. 7). OUT/UKN can be adjacent to any other outside region.

#### UNKNOWN: Unknown

The label UNKNOWN is selected when the operator does not know at all where the satellite is (due to a lack of data for example or very complex region), and when it is not even sure that the satellite location is inside (IN/UKN) or outside (OUT/UKN) the magnetosphere. Typically it can follow N/A at large distances when the spacecraft has its perigee in the magnetospheric tail. An illustration is given in Fig. 7 (right panels).

#### N/A: No data available

When no data at all are available, the label N/A is selected (see right panels in Fig. 7).

## Data Records

The dataset is available at Figshare repository^43^ and at the CSA^9^. The GRMB data files are saved in the Cluster Exchange Format (CEF), which is the default file format for archiving the Cluster data. CEF files can be read with any text editor ASCII-compatible and include a header containing all the information required to read and interpret the data. The CSA website hosts a full description of the CEF file format (https://www.cosmos.esa.int/web/csa/documentation). The data records start after the line containing the “*DATA*_*UNTIL* = *END_DATA*” text and they can be read and interpreted with the following description. There is one file available per spacecraft and each file covers the 2001-2007 time period. These files can be accessed through a repository^43^ or directly downloaded^44^. The spacecraft identifier is the two first characters of the filename (C1, C2, C3, or C4). The user can download any other time interval at the CSA^9^.

**Fig. 8:**
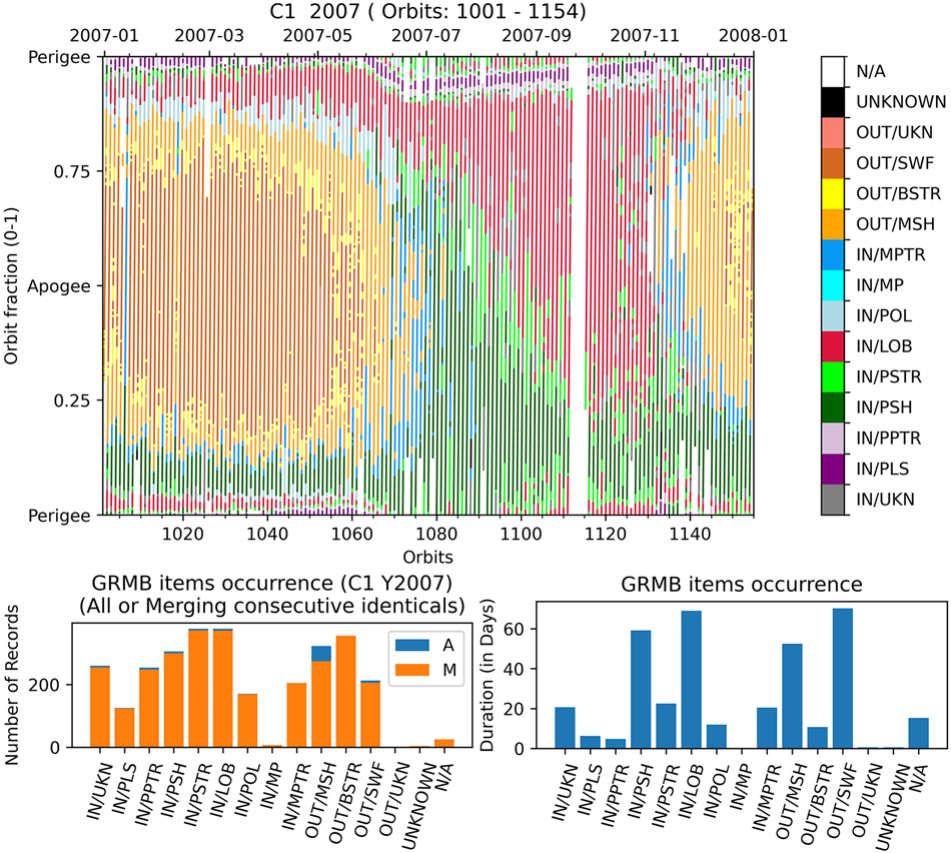
Top: Location of the Cluster-1 spacecraft in year 2007 (orbits 1001 to 1154) according to the GRMB dataset. Bottom: number of hits (left) and duration spent by the spacecraft (right) for each label.

Seven variables are enclosed and coma separated in the GRMB dataset CEF files in the following order: *time_tags*, *location_label*, *location_code*, *quality_location_label*, *quality_location_code*, *inbound_vs_outbound*, and *crossing_complexity*. The variable description comes along with illustrative figures produced for Cluster-1 during year 2007. The data are plotted as a function of time in x-axis and the orbit fraction in y-axis (see Figs. 8 top, 10 right, 11 and 12 right). This representation is also known as Bryant plots in the frame of the Cluster mission^45^.

### *time_tags*, *location_label* and *location_code* variables

Each file entry, a record, is associated with a region label (*location_label* variable, 2-digit entry) a region index (*location_code* variable, string entry, “” delimited) listed in Table 1 and a time interval (*time_tags* variable, 2 Universal Time entries, / separated). Time entry format is *YYYY-MO-DDTHH:MM:SSZ*, where *YYYY* stands for the year, *MO* for the month, *DD* for the day, *HH* for the hour, *MM* for the minutes, and *SS* for the seconds.

The start time (first value of the *time_tags* variable) has been selected by the operator. The end time (second and last value of the *time_tags* variable) has been selected by the operator as the start time of the next record. This ensures a continuous coverage of the labelling. The label and index have been listed in Table 1.

These three variables locate the spacecraft in the geomagnetic space as seen in the top panel of Fig. 8. The solar wind region (SWF, in brown) is crossed from December to May. It is separated from the magnetosheath (MSH, in orange) by the bow shock (BSTR, in yellow). The exit of the magnetosphere occurs before the apogee through the magnetopause (MP, in light blue, not observed during this year and for this satellite) or the magnetopause transition region (MPTR, in cyan) into the plasmasheet (PSH, in dark green). The entry in the magnetosphere occurs after apogee through the polar regions (POL, in blue) into the lobes (LOB, in crimson). When the spacecraft stops crossing the solar wind it spends most of the time in the plasmasheet transition region (PSTR, in light green), for instance in July. Then the duration spent in LOB is larger from July to November. The plasmapause transition region (PPTR, in pink) and the plasmasphere (PLS, in violet) are commonly observed close to the perigee all along the year. This description is matching the expectations of the Cluster location.

Only a few regions inside the magnetosphere are unidentified (IN/UKN, in gray). One can note three orbits without data (N/A, in white) at the end of September. Unknown regions (UNKNOWN, in black) and unknown regions outside the magnetosphere (OUT/UKN, in salmon) are not present for this year.

The bottom plots in Fig. 8 help to quantify those observations. Considering the time spent in each region (right panel), we note that the spacecraft is located most of the time in the PSH, the LOB, the MSH and the SWF (more than 250 days all together in 2007). When considering the number of times that a region is crossed by the spacecraft (number of records per item, left panel), the occurrence is more evenly distributed between the items (very few MP, OUT, UNKNOWN, N/A). This is particularly striking for the PLS and the PPTR which are encountered one and two times respectively per orbit for a short period. One can note that there are less SWF records than BSTR records: There are roughly two BSTR (inbound and outbound) for one SWF.

The same entry can appear several times in a row. If we merge those consecutive records (orange distribution), the number of records changes mainly for the MSH, probably due to the turbulent aspect of this region (see Section *crossing_complexity*).

### *quality_location_label* and *quality_location_code* variables

*quality_location_label* is a 32-digit variable that contains all the panels that were displayed at the selection time (region entry time). Each two digits correspond to a CSA product identifier (first column in Table 2). *00* is the fill-value (not a CSA product). A view can display up to 8 panels and two views can be simultaneously displayed. In total a maximum of 16 panels can be available. The standard number of active panels is 12. This variable is not intended for a scientific purpose. In case of doubt about a boundary, the user can check which panels were displayed at the selection time.

*quality_location_code* (single digit entry) provides the quality of the panel combination (*quality_location_label*) for a given entry. It can take 5 different values (0, 1, 2, 3, or 4). For each GRMB label we define in Table 3 the prime products necessary to perform a correct identification. A prime product can be taken from one or several CSA products. Prime products dependent on more than one CSA product are listed in Table 4. The full name of the CSA products can be found in Table 2. For example ‘density’ means either CIS/HIA, CIS/CODIF, WHISPER or PEACE density measurements. To reduce the number of panels to be displayed at a single time, the selection tool displays the CSA product with the best data coverage.

**Table 3:** List of the prime products for each GRMB item.

Index	Short Name	Prime Products
1	IN/UKN	‘Epart’, ‘PAhigh’, ‘Bx’, ‘Btot’, ‘Vx’
2	IN/PLS	‘E2whisper’, ‘Nwhisper’, ‘POT’
3	IN/PPTR	‘E2whisper’, ‘Nwhisper’, ‘POT’
4	IN/PSH	‘Epart’, ‘PAhigh’, ‘PAlow’
5	IN/PSTR	‘Epart’, ‘PAhigh’, ‘PAlow’
6	IN/LOB	‘Epart’, ‘Bx’, ‘Btot’, ‘PAhigh’,’PAlow’
7	IN/POL	‘Epart’, ‘PAhigh’, ‘Bx’, ‘Btot’, ‘Vx’
8	IN/MP	‘Epart’, ‘PAhigh’, ‘Bx’, ‘Btot’, ‘Vx’
9	IN/MPTR	‘Epart’, ‘PAhigh’, ‘Bx’, ‘Btot’, ‘Vx’
10	OUT/MSH	‘density’, ‘Vx’, ‘Btot’, ‘waves’, ‘Ecis’
11	OUT/BSTR	‘density’, ‘Vx’, ‘Btot’, ‘waves’, ‘Ecis’
12	OUT/SWF	‘density’, ‘Vx’, ‘Btot’, ‘waves’, ‘Ecis’
13	OUT/UKN	‘Epart’, ‘PAhigh’, ‘Bx’, ‘Btot’, ‘Vx’
14	UNKOWN	‘Epart’, ‘PAhigh’, ‘Bx’, ‘Btot’, ‘Vx’
15	N/A	—

**Table 4:** Detail of the prime products.

Prime product	Short name
Btot	Btot
Bx	Bx
Density	N_hia
	N_peace
	N_whisper
	N_codif
Nwhisper	Nwhisper
POT	POT
Ecis	Ehs_hia
	Eh_codif
Epart	Ehs_hia
	Eh_codif
	Eall_peace
PAhigh	PAhigh_hia
	PAhigh_codif
	PAhigh_peace
PAlow	PAlow_hia
	PAlow_codif
	PAmid_peace
Vx	Vx_hia
	Vx_peace
	Vx_codif
Waves	B2_staff
	E2_whisper
E2_whisper	E2_whisper

The *quality_location_label* variable value is based not only on the display of the products at the selection time but also on the availability of these products as empty plots are considered as missing. The *quality_location_code* value depends on the number of prime products available at the selection time (see Table 5). One can note that for the items with only 3 prime products *quality_location_code*  = 2 is not possible.

**Table 5:** Description of the *quality_location_code* variable.

Value	Label	Description
0	Bad	No prime product is available
1	Low	1 prime product is available
2	Fair	More than 1 product but less than all-1 prime products are available
3	Good	All-1 prime products are available
4	Top	All prime products are available

**Fig. 9:**
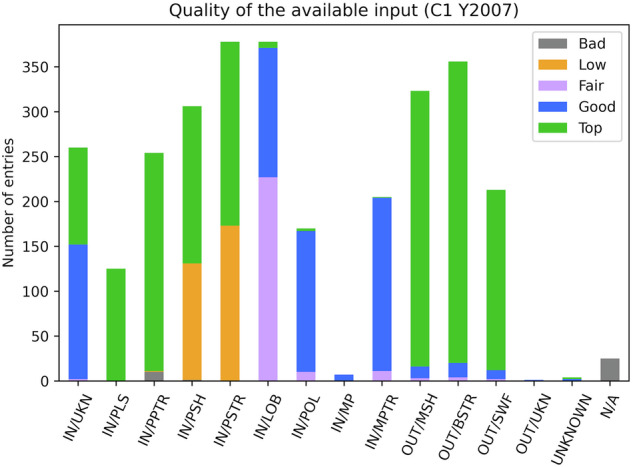
Quality of the available dataset for the boundary selection for Cluster-1 in 2007 (orbits 1001 to 1154): number of hits for each quality value (*Bad*, *Low*, *Fair*, *Good* and *Top*) and for all 15 GRMB items.

Figure 9 presents the *quality_location_code* value in 2007 for C1. *Bad* values are found as expected for the N/A entry. Few PPTR labels display also such value. In such cases it rather results from a permutation between different views to identify the boundary. The operator has probably checked the plasmapause with the prime parameters but he also double-checked with another view. PSH and PSTR take *Top* or *Low* values, as their prime products rely on particle data. *Top* values are obtained when the products are available and *Low* when some are missing. Low values can also result from the selection of the boundary by comparison with other views (*IN-Plasmasphere* and *IN-Magnetosphere*, for example). The LOB, POL, MP, and MPTR display *Fair* or *Good* quality values. The one or two missing products result for one part from the unavailability of certain products and for the other parts from the difficulty to get all the prime products on a single view. Overall the quality of the boundary selection is satisfactory. 56% of the boundaries are selected with a *Top* value and 80% with a *Top* or a *Good* value.

A dataset user shall not restrict the data quality to the top values. In case of down-selection based on this variable, we consider that all quality values above 2 (*Fair*, *Good* and *Top*) lead to a consistent selection of regions.

### *inbound_vs_outbound* variable

**Fig. 10:**
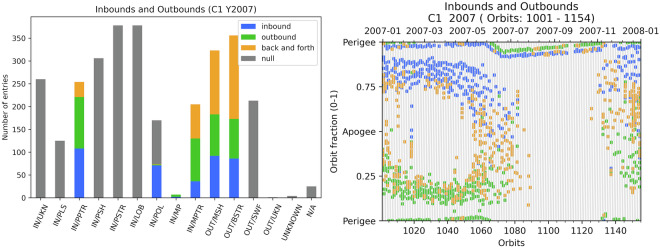
Left: Bound distributions in the available dataset for the boundary selection for the Cluster-1 spacecraft in year 2007: number of hits for each bound value and for each GRMB item. Right: *inbound_vs_outbound* for PPTR, POL, MP, MPTR, MSH, and BSTR.

We define a single inbound value for each record (*inbound_vs_outbound* is a single digit entry that can take 5 different values (0, 1, 2, 3 or 4). Our understanding of an inbound is that the preceding region is in average farther from the Earth and the following one closer. This variable is automatically set based on the preceding and following record. It is computed for the following regions: PPTR, POL, MP, MPTR, MSH, and BSTR. The combinations for the inbound (*inbound_vs_outbound*  = 1, in blue in Fig. 10) and outbound (*inbound_vs_outbound*  = 2, in green in Figure 10) are presented in Table 6. For example, for the three successive labels SWF-BSTR-MSH, BSTR is defined as an inbound. Same situation for SWF-BSTR-BSTR (in case of consecutive multiple entries). Any other combination than the ones listed in Table 6 leads to *inbound_vs_outbound*  = 3 for MP, MPTR MSH, BSTR and PPTR (in orange in Fig. 10).

**Table 6:** Definition of inbound and outbound crossings for each label, based on the preceding and following labels.

Label	Inbound		Outbound	
	Preceding	Following	Preceding	Following
BSTR	SWF	MSH, BSTR	MSH	SWF, BSTR
	BSTR*	MSH	BSTR	SWF
MSH	BSTR	MP, MPTR, POL, MSH	MP, MPTR, POL	BSTR, MSH
	MSH*	MP, MPTR, POL	MSH	BSTR
MP, MPTR, POL	MSH, OUT/UKN	MP, MPTR, POL, LOB, PSTR, PSH, PLS, PPTR, IN/UKN	LOB, PSTR, PSH, PLS, PPTR, IN/UKN	MP, MPTR, POL MSH, OUT/UKN
	MP, MPTR, POL	LOB, PSTR, PSH, PLS, PPTR, IN/UKN	MP, MPTR, POL	MSH, OUT/UKN
PPTR	LOB, PSTR, PSH	PLS, PPTR	PLS	LOB, PSTR, PSH, PPTR
	PPTR	PLS	PPTR	LOB, PSTR, PSH

Note that the same label can happen more than once in a row.

For the other labels (IN/UKN, PLS, PSH, PSTR, LOB, mid-altitude POL, SWF, OUT/UKN, UNKNOWN and N/A): *inbound_vs_outbound*  = 0 (see gray bars in Fig. 10, left panel). Note the difference between the 0 value (there is no bound definition for the item) and the 1 value (the kind of bound is not defined for this entry).

The overall inbound and outbound number of hits is roughly similar (Fig. 10, left panel). The location of the different *inbound_vs_outbound* is in agreement with the spacecraft orbit (Fig. 10, right panel). At the item level, MPTR are in majority outbound crossings, whereas POL are mainly inbound crossings. This is in accordance with Fig. 8 where the MPTR entries are observed before apogee and POL after.

The other type of bound usually results from back and forth motion of the boundary with respect to the spacecraft (in orange in Fig. 10). As an illustration the MSH-BSTR-MSH combination leads to a *inbound_vs_outbound*  = 3 for the BSTR label. A secondary cause for these values is to have a preceding or following N/A or unknown (IN/UKN, OUT/UKN, UNKNOWN) region.

**Fig. 11:**
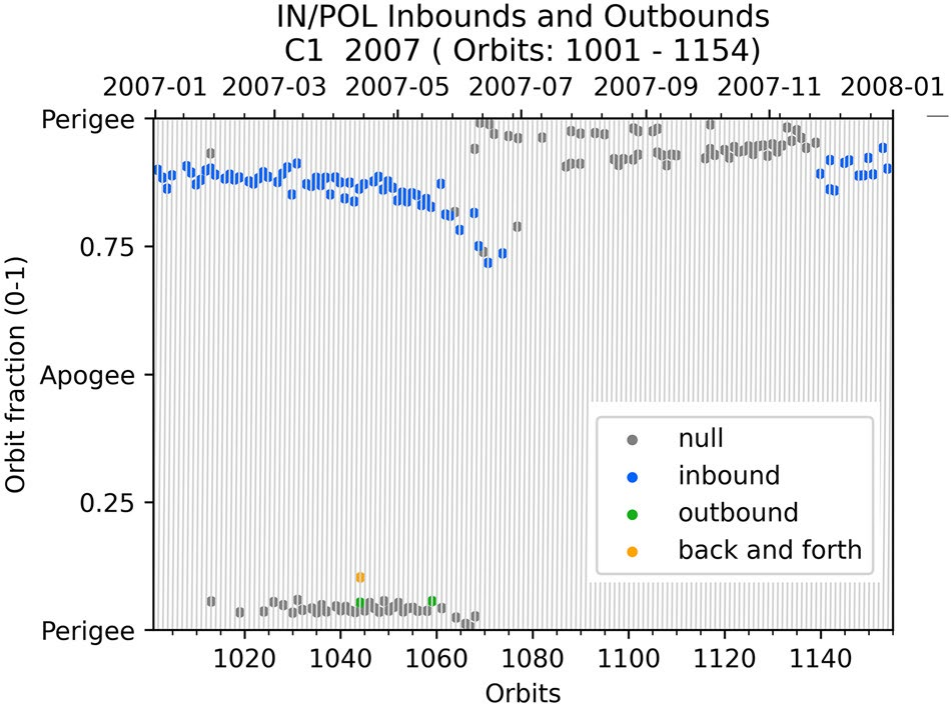
Mid-altitude (in blue) vs distant (all other colors) polar region crossings.

POL is a specific item for *inbound_vs_outbound*. If the preceding and following regions form a combination listed in Table 6, it is considered as an inbound or outbound. If not, a *inbound_vs_outbound*  = 3 happens when either the preceding or following item is one of [MP, MPTR, MSH, OUT/UKN], a *inbound_vs_outbound*  = 0 otherwise. This offers a quick way to separate distant polar region region crossings (*inbound_vs_outbound* is 1, 2 or 3) and mid-altitude polar region crossings (*inbound_vs_outbound*  = 0) as illustrated in Fig. 11.

We emphasize here that the bound in the GRMB dataset is defined at the region level and not at the boundary level. At the boundary level only two entries are needed to define the bound (SWF followed by BSTR means an inbound boundary crossing).

### *crossing_complexity* variable

This variable (*crossing_complexity* is a single digit entry) takes only two values (0 or 1). The default value 0 means that the region displays the expected properties during the crossings (simple crossing). If the observations depart from the expectations, the operator can manually set this variable to 1 (complex crossings). It usually indicates that a mixture of properties is observed: Complex PSTR probably means that LOB properties alternate with plasmasheet-like observations. When these properties are fully mixed and when there is a doubt for the selection of the item, the operator should rather select IN/UKN, OUT/UKN, or UNKNOWN. For these three labels, the default 0 value means lack of data for a proper identification and a 1 value means that data are available but there is no label corresponding to the observed properties.

During the selection, the operator usually follows that specific rule: For BSTR and MPTR, select *crossing_complexity*  = 1 when several bow shock or magnetopause crossings are seen during that time interval. *crossing_complexity*  = 1 can correspond to a mix of properties observed inside a region. For example when weak particle fluxes or short scale PSTR signatures are observed inside a LOB entry. *crossing_complexity*  = 1 can also correspond to a change of property within a region, for example when switching inside the magnetosheath to a turbulent regime, or if the foreshock signatures are strong compared to the nearby solar wind, the operator shall raise this flag. When the density evolution is not well seen at the perigee, *crossing_complexity*  = 1 shall be set for PPTR and PLS. *crossing_complexity* is a kind of manually set quality flag.

**Fig. 12:**
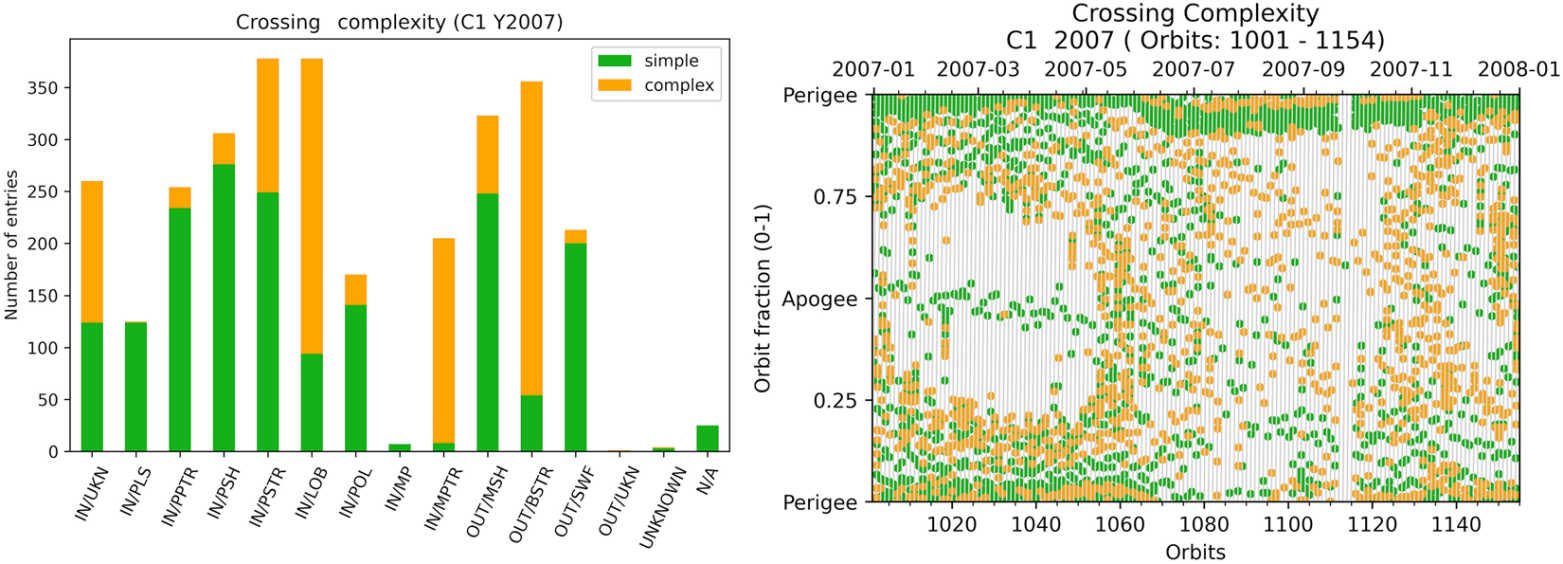
Left: Crossing complexity distributions in the available dataset for the boundary selection for Cluster-1 in 2007: number of hits for each bound value and for each GRMB item. Right: *crossing_complexity* for PPTR, POL, MP, MPTR, MSH, and BSTR.

The main interest of this is for global studies. Keeping only 0 values insures a better region qualification, or single boundary crossings. Looking at Fig. 12 there is no specific location corresponding to one of the *crossing_complexity* value. PLS, PPTR, PSH and SWF are usually clean regions.

### Cluster location in geospace

**Fig. 13:**
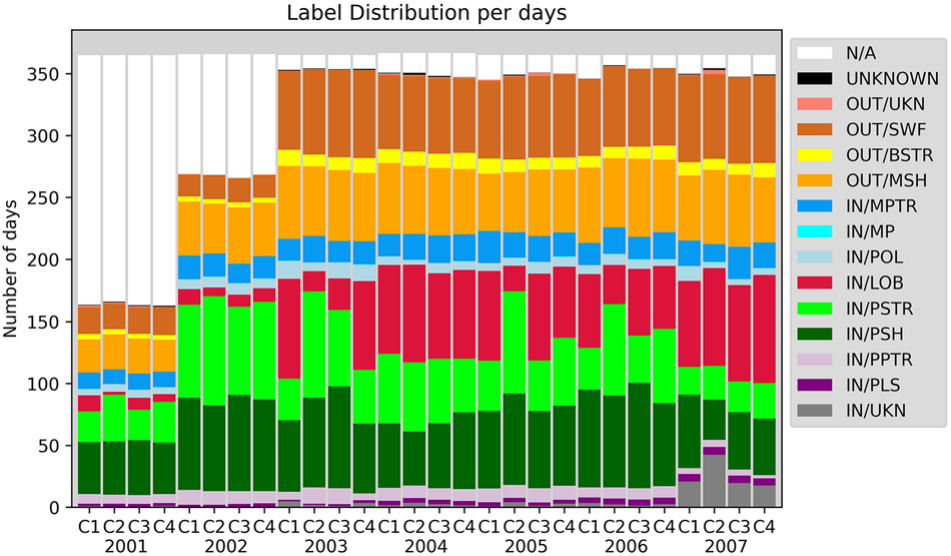
Number of days per year per spacecraft for each GRMB label over years 2001-2007.

Figure 13 summarizes the location in number of days of each Cluster spacecraft between year 2001 and 2007. The first two years contain a larger proportion of N/A data as the mission was still operating in a non-continuous observation mode. The number of days spent in the items is remarkably stable over time and from one spacecraft to the other, LOB and PSTR excepted. In general the LOB duration is shorter for C2 than for the other spacecraft. This comes from the lack of ion measurements onboard C2. This is not true for the years 2004 and 2007 that were processed with more comparisons between spacecraft during the region selection. This was however too time consuming for being done for the remaining years. The second feature is the increased number of IN/UKN region in 2007. This has two causes: the operator is more confident after a few years of survey that it is difficult to choose a region, the second one is that the data of some instruments were missing more often than before.

This figure confirms what was presented in the previous sections. The LOB, PSTR, IN/UKN and to a lower extent PSH and POL are the most difficult to separate. We can mention here that the next phase of this project, which will consist in delivering a dataset for the full mission duration, will contain a package to make these regions more homogeneous. The feedback of the operators is making us confident that we can improve the labeling of these regions.

Table 7 provides the time percentage covered by each label for each spacecraft during three time periods: year 2001, year 2002, and years 2003-2007. In year 2001 and 2002 the main label is N/A which corresponds to the part-time operation of the mission during these years to guarantee the observations in the key regions in the nominal phase of the mission.

**Table 7:** Time percentage covered by each of the 15 labels for each Cluster spacecraft in year 2001, 2002, and 2003-2007.

GRMB	2001				2002				2003-2007			
label	C1	C2	C3	C4	C1	C2	C3	C4	C1	C2	C3	C4
IN/UKN	0.3	0.2	0.1	0.3	0.1	0.1	0.1	0.2	1.7	3.0	1.4	1.5
IN/PLS	0.6	0.6	0.7	0.7	0.5	0.5	0.6	0.7	1.0	1.1	1.1	1.1
IN/PPTR	2.1	2.0	1.9	2.0	3.3	3.0	2.8	2.7	2.2	2.7	2.6	2.1
IN/PSH	11.5	11.8	12.2	11.4	20.3	18.9	21.2	20.3	17.0	16.2	18.0	16.2
IN/PSTR	6.8	10.4	6.7	9.0	20.6	24.2	19.5	21.6	10.2	17.9	11.9	12.6
IN/LOB	3.6	0.7	2.6	1.7	3.5	1.9	2.6	3.0	19.3	12.3	16.1	18.4
IN/POL	1.4	1.7	1.8	1.5	2.0	2.3	2.4	2.0	2.6	1.7	2.3	2.1
IN/MP	0.0	0.0	0.0	0.0	0.0	0.0	0.0	0.0	0.0	0.1	0.1	0.1
IN/MPTR	3.7	3.2	3.6	3.4	5.2	5.1	4.3	4.9	5.5	5.4	5.7	5.6
OUT/MSH	7.3	7.7	7.7	7.0	11.9	11.0	12.4	11.8	15.1	15.1	15.7	14.8
OUT/BSTR	1.2	1.2	1.1	1.1	1.1	1.0	1.2	1.1	3.1	2.7	2.7	3.2
OUT/SWF	5.9	5.6	5.8	6.2	4.9	5.3	5.4	5.1	17.4	18.0	18.0	18.1
OUT/UKN	0.4	0.3	0.3	0.1	0.1	0.0	0.0	0.0	0.2	0.3	0.2	0.1
UNKNOWN	0.1	0.1	0.2	0.3	0.0	0.0	0.0	0.0	0.1	0.3	0.1	0.1
N/A	55.2	54.5	55.3	55.4	26.5	26.8	27.4	26.7	4.5	3.4	4.0	3.9

In years 2003–2007 five labels are almost found in a similar proportion, between 15% and 20%: LOB, PSTR, PSH, MSH, and SWF, corresponding to about 80% of the time. Even if PPTR and PLS are crossed close to the perigee where the spacecraft velocity is higher, these labels cover more than 3% of the time, a proportion similar to POL. More time is spent in the MPTR (6%) than in the BSTR (3%) indicating that the magnetopause boundary is not as sharp as the bow shock, and/or that the orbits make the spacecraft skimming during a longer time close to the magnetopause than to the bow-shock. One can note that the label MP corresponds to a very small proportion of time: sharp magnetopause crossings are not frequent and last short by definition.

One can note that on average the spacecraft location is not available less than 5% of the time (N/A and UNKNOWN labels). Finally the proportion of IN/UKN (2%) is larger than OUT/UKN, showing the difficulty to label the regions inside the magnetosphere compared to the outside regions.

These numbers are remarkably similar from one spacecraft to the other, which is an expected result due to the similar orbit of the spacecraft. It demonstrates the consistency of the GRMB dataset. Only C2 marks a clear difference with the three other spacecraft for the PSTR and LOB regions, because of the missing ion data onboard C2.

## Technical Validation

We validated our dataset over the years 2001-2007 for the four spacecraft. These years cover all the GRMB labels and include events from several selected reference datasets: 1 dataset for the BSTR label^41^, 1 for the PPTR and PLS labels^46^, 1 for the POL label^35^, 1 for the MP and MPTR labels^47^ and 1 for the PSH, PSTR and LOB labels^48^.

### Comparison with “by-eye” reference lists

The GRMB labels at the bow shock times given in Kruparova, *et al*.^41^ are listed in Table 8. More than 95% of the bow shocks are found in a BSTR region in the GRMB dataset. This high degree of consistency validates our methodology. There are of course much more BSTR entries in the GRMB dataset. In few cases, bow shocks are found in SWF or MSH regions, possibly related to a short back-and-forth crossing. The 3 UNKNOWN labels are related to unclear shock crossings. The single N/A entry is related to lack of data for this entry and the single MPTR entry is related to an operator mistake.

**Table 8:** GRMB location of the bow shocks listed in Kruparova, *et al*.^41^ in years 2001-2007 for all 4 Cluster spacecraft.

	BSTR	MSH	SWF	UNKNOWN	N/A	MPTR
C1	448 (98%)	1	8	1	—	—
C2	441 (96%)	5	10	2	—	—
C3	443 (97%)	2	11	—	1	1
C4	438 (96%)	4	16	—	—	—

For the plasmapause, we checked if the timing of the plasmapause crossings listed in Darrouzet, *et al*.^46^ are found in a PPTR entry. The plasmapause reference crossings are found in 98% in a PPTR label. In the mismatching cases (3 PLS, 1 N/A), a PPTR entry is found within less than 10 minutes of the reference time.

We checked if the timings of the magnetopause crossings dataset built by Fuselier, *et al*.^47^ available via our dataset^9^ (or at https://www.cosmos.esa.int/web/csa/crossings-techniques), are found in MPTR, MP or POL labels of the GRMB dataset. There is an agreement in 3817/4523 entries in years 2001 to 2007 (84% of agreement). This value is a bit lower than for BSTR and PPTR comparisons, because the reference list in that cases is not restricted to the most convincing crossings. Logically the main source of mismatches are the regions located close to the magnetopause: MSH (321 mismatches, 7% of the reference crossings) and PSTR-PSH-IN/UKN (267 mismatches, 6%). Many discrepancies result from a short timing difference (less than 10 minutes). In that case, it means that we started the magnetopause label a bit too late or ended it too early, possibly due to the manual click selection in the 6h view. Another large proportion of events corresponds to events where the spacecraft are seen in the magnetosheath and there is no way a posteriori to see that there was a magnetopause: the product and the time resolution of the plots do not allow us to retrieve all the reference results. Finally there is also quick back-and-forth crossings that are not caught via our methodology. Surprisingly there are few BSTR labels (23 mismatches, 0.6%) that can correspond to operator mistakes and/or to a different data appreciation. In 2% of the reference crossings (95 mismatches) there were no data available in the region selection tool.

In the polar regions, we checked if the timing of the cusp crossings listed in Pitout, *et al*.^35^ are found in a POL label in the GRMB dataset. The 92 % match is a good result (see Table 9). Half of the mismatches results from small timing differences (the operator starts the POL region a bit later, for example). In three cases there is a difference of interpretation (OUT/MSH), in one case there is a wrong click from the operator (IN/MP) and in a few cases of mixed observed properties the operator could have selected POL or IN/UKN (and not PSH or PSTR). Of course, the GRMB dataset contains more POL labels than the ones listed in the table.

**Table 9:** GRMB location of the cusp listed in Pitout, *et al*.^35^ in years 2001-2007 per Cluster spacecraft.

	POL	MSH	PSH	PSTR	MP
C1	81 (93%)	1	4	1	—
C3	78 (92%)	1	4	2	—
C4	83 (93%)	1	3	1	1

### Comparison with “automatic classification” reference lists

The ECLAT^48^ and GRMB datasets are built in a different way. Firstly, the ECLAT dataset is limited in time and space, covering the nightside of the magnetosphere during the years 2001-2009, while the GRMB offers a continuous coverage. Secondly, the time resolution of the ECLAT dataset is a few seconds to be compared to the 20 minutes of the GRMB dataset. Thirdly, the ECLAT dataset is built by an automatic procedure and the GRMB dataset results from a manual selection. Finally the input dataset and the output labels are different in the two sets. Therefore, there is no reason for finding a good match between the two datasets. However, when setting a correspondence between the labels of the two datasets a few things can be learned from a comparison.

We compare the ECLAT and GRMB dataset regions in the following way. ECLAT undefined regions (*UR*) are compared with the GRMB unknown region (UNKNOWN and IN/UKN). Lobes (*NN*_*LOBE* and *SS*_*LOBE*) ECLAT regions are compared with GRMB lobe region (LOB). Boundaries (*NN*_*BR* and *SS*_*BR*) ECLAT regions are compared with GRMB plasmasheet transition regions (PSTR). Plasmasheet related ECLAT regions (*NN*_*OPS*, *SS*_*OPS*, *NN*_*IPS*, *SS*_*IPS*, *NN*_*NSR*, *SS*_*NSR*, 00_*NSR*) are compared with GRMB plasmasheet regions (PSH). Then we look for the GRMB label of each ECLAT record. The results are presented in Table 10.

**Table 10:** GRMB-ECLAT comparisons in years 2001-2007 for the four spacecraft.

	ECLAT	Match	GRMB							
	PSH		PSTR	PSH	LOB	IN/UKN	MPTR	MSH	UKNs	N/A
C1	42758	29437 (69%)	11434	24937	197	356	1319	—	—	15
C2	38435	30829 (80%)	6438	30829	14	753	328	—	5	68
C3	54095	45061 (83%)	7776	45061	175	252	756	—	23	52
C4	48596	36058 (74%)	11136	36058	149	294	930	—	2	27
	PSTR		PSTR	PSH	LOB	IN/UKN	MPTR	MSH	UKNs	N/A
C1	43526	21666 (50%)	21666	15395	5635	764	25	—	—	41
C2	28431	9740 (34%)	9740	17158	1005	150	304	—	22	52
C3	58911	17248 (29%)	17248	38353	2851	105	306	—	—	48
C4	53880	25881 (48%)	25881	21887	5424	114	466	—	4	104
	**LOBE**		**PSTR**	**PSH**	**LOB**	**IN/UKN**	**MPTR**	**PPTR**	**UKNs**	**N/A**
C1	25943	6261 (24%)	14805	4595	6261	8	218	—	—	6
C2	15017	1574 (11%)	6892	6320	1574	32	127	—	31	60
C3	29462	3298 (11%)	12280	13692	3298	28	103	1	28	60
C4	30604	6816 (22%)	17140	6380	6816	31	115	—	31	122
	**Unknown**		**PSTR**	**PSH**	**LOB**	**PLS**	**MPTR**	**MSH**	**UKNs**	**N/A**
C1	7493	206 (3%)	1828	2647	639	61	1706	406	11	195
C2	3524	269 (8%)	966	1034	422	70	759	4	97	172
C3	5099	208 (4%)	678	1667	436	64	1716	330	25	183
C4	6978	272 (4%)	1282	2066	1394	64	1466	434	65	207

Number of GRMB entries for each of the four ECLAT categories (PSH, PSTR, LOBE, Unknown).

Our first comment is that the overall percentage of agreement is lower than for the other reference lists, because of the previously mentioned differences between the two datasets. We also note the high variability found in the number of ECLAT records between two spacecraft.

An important result is that almost all (>98%) the valid ECLAT records (not classified as undefined in the ECLAT dataset) are found in one of the three PSH, LOB and PSTR GRMB labels. The best match is found for the PSH labels where there is about 75% matches between the two different datasets. The mismatches are mainly found in PSTR which is a satisfactory result.

Then the boundaries of the ECLAT regions are in a fair agreement with the GRMB PSTR label (≈40%). An important result is that more ECLAT boundary entries are classified PSH rather than PSTR in the GRMB dataset. The panels available for the visual detection do not probably allow the distinction made in ECLAT. LOB is only found in a small fraction (<10%) of the ECLAT records. This is in adequation with the GRMB methodology: in case of quickly changing properties, the main region will be set. It is thus no wonder that the LOB label contains some ECLAT boundary events.

The lobe ECLAT labels display a low match with the LOB GRMB label (≈15%). However, the LOB GRMB labels are more present in this ECLAT category than in the other ECLAT categories. This is indicative that the GRMB lobe label corresponds effectively to the lobes but that some part of the lobes are missing, probably the part where the properties are quickly changing and where some particle fluxes convinced the operator to opt for a PSTR entry.

As a first conclusion, the LOB GRMB label contains probably clean lobe entries and PSH GRMB label contains probably clean plasmasheet events. To get an optimum coverage of the lobe (or the plasmasheet) it is advisable to add PSTR to LOB (or PSH) and make an ad hoc down-selection.

The undefined ECLAT regions (Unknown in Table 10) are usually found in well-identified GRMB regions and are marginally classified as UNKNOWN or IN/UKN. It is worth noting that a large part of these ECLAT entries are classified as MPTR, MSH or PLS, which makes sense.

The comparison with the ECLAT dataset shows that the methodology is quite accurate for identifying the regions located inside the magnetosphere even if the GRMB dataset is less detailed than other datasets due to a lower time resolution. It can be noted that there are much less undefined regions in GRMB than in ECLAT. Adding to this the results of the previous section (accurate detection of the polar cusp, the magnetopause, the plasmapause and the bow-shock), the methodology defined for building the GRMB dataset is validated.

### Comparison with Bryant plots

The Cluster’s Bryant plots (https://csa.esac.esa.int/csa-web/#bryant) have been used for planning the operations during the active phase of the mission and represent the predicted positions of the modeled magnetopause^49^ and bow shock^50^ as a function of the orbit phase and the orbit numbers. We draw a blue symbol for each BSTR record at the spacecraft location in the center of the interval. Similarly, we draw a red symbol for each MP and MPTR record. We did not include POL label (that also includes some magnetopause crossings) because this label can extend close to the perigee and make the comparisons more difficult.

**Fig. 14:**
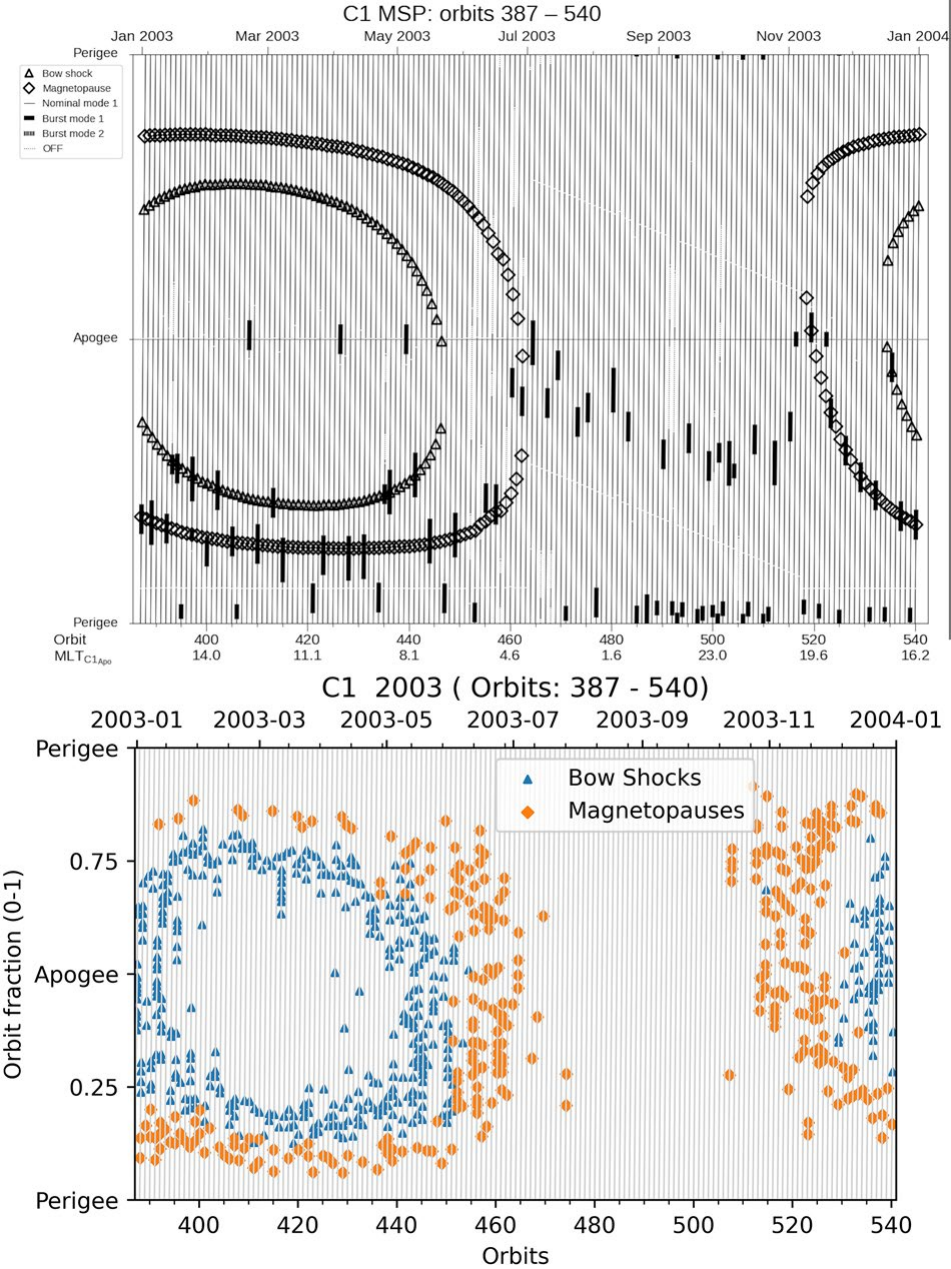
Cluster-1 location in 2003. Top: Bryant plot of the predicted magnetopause (diamonds) and bow-shock (triangles) crossings. Bottom: Center time of the MPTR, MP and BSTR labels of the GRMB dataset.

Figure 14 presents comparisons for the year 2003: on the top the original Bryant plots and on the bottom the GRMB Bryant plot. At large scale, the representation is very similar. The bow shock and magnetopause locations are found for a given orbit at the same orbit phase. The number of these boundary crossings drastically decreases between mid-July and mid-November. In January-April the orbit inclination makes the magnetopause crossings closer to the bow shock in the first part of the orbits (phase < 0.3) than in the last part. This feature is also found in the GRMB plots. Few differences are also seen. In the original Bryant plots (top) the boundaries are almost forming a continuum.

In the GRMB Bryant plot (bottom), the distribution is not continuous anymore: the location changes from one orbit to another, reflecting the non-stationarity of the interplanetary medium. We found less magnetopause crossings than expected when Cluster is approaching its perigee (upper part of the Bryant plots). This is because in this region there are also many cusp crossings that are labelled as POL (not included here, as explained here above).

As a conclusion for this section, our dataset reproduces the expected location of the bow shock and the magnetopause in Cluster orbits. Based on comparison with the Bryant plots, the GRMB dataset can be considered as technically validated.

### Comparison between region properties

The GRMB dataset can be used to perform statistical studies or identify interesting events in specific regions. This section presents examples of application of the GRMB dataset and gives guidance on how to use at best the GRMB dataset based on user needs. Two examples of application of the GRMB dataset for Cluster-1 in 2007 are discussed below, the first one for the magnetosphere and the solar wind, the second one for regions inside the magnetosphere. A special emphasis is put on the differences between regions and transition regions. Understanding these differences is key to get the best from the GRMB dataset.

The GRMB output files provide the location of the Cluster spacecraft in geomagnetic space. Each region is associated with a region label (*location_label*) and region index (*location_code*), each of which can be used to identify the region in which the satellite is located according to the GRMB dataset. The GRMB output files provide also a start time (corresponding to Cluster entry in a region) and a stop time (corresponding to Cluster exit of a region) for each region crossed by the Cluster spacecraft. The GRMB start and stop times can be used to download Cluster data for a specific region from the CSA archive. In the following examples, the CSA data are downloaded using command lines provided by ESA (https://www.cosmos.esa.int/web/csa-guide/matlab-idl-or-python)^51^ for the time periods in the region of interest. With this method it is possible to extract Cluster data for specific regions without the need to store Cluster data locally.

#### Comparison between region properties: Solar wind and Magnetosphere

**Fig. 15:**
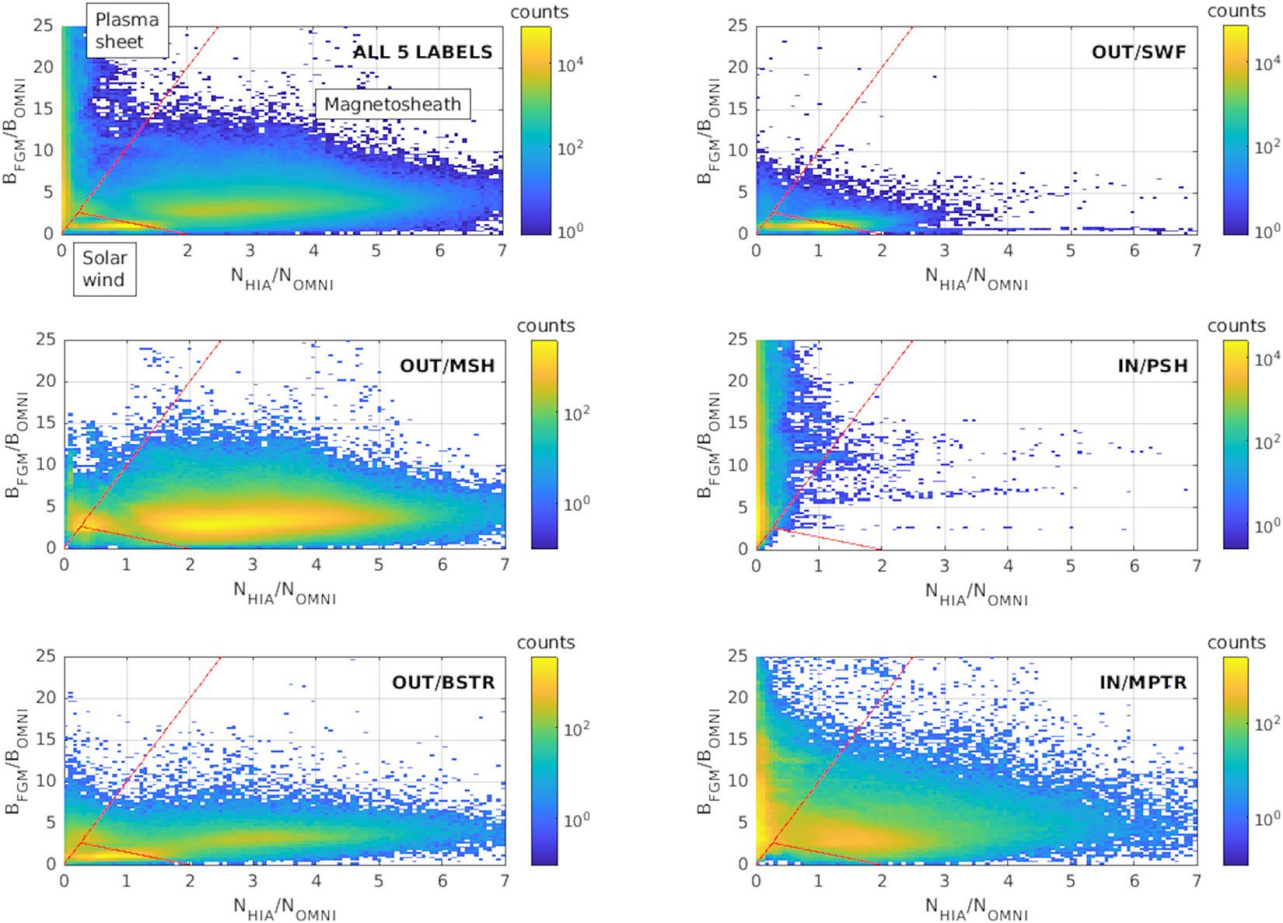
Two-dimensional histograms of the magnetic field measured in 2007 by Cluster-1 divided by the IMF magnitude as a function of the density measured by Cluster-1 divided by the solar wind density. The density is obtained from the CIS/HIA experiment and the magnetic field from the FGM instrument. The regions are identified according to the GRMB dataset. Top left: for all regions and transition regions (OUT/SWF, OUT/MSH, IN/PLS, OUT/BSTR and IN/MPTR); Top right: for the solar wind (OUT/SWF); center left: for the magnetosheath (OUT/MSH), center right: for the plasmasheet (IN/PLS); bottom left: for the bow shock transition region (OUT/BSTR); bottom right: for the magnetopause transition region (IN/MPTR). The solar wind density and the IMF magnitude are shifted to the bow shock and obtained from the OMNI database^52^. Cluster data have been obtained from the Cluster Science Archive (CSA). The red lines correspond to separatrices between the magnetosphere, the solar wind and magnetosheath as defined by Nguyen, *et al*.^6^.

Figure 15 shows results for Cluster-1 during the year 2007. It represents the distribution of data in the density - magnetic field plane for three GRMB regions (the solar wind foreshock (SWF), the magnetosheath (MSH) and the plasmasheet (PSH)) and two transition regions (the bow shock transition region (BSTR) and the magnetopause transition region (MPTR)). The density plotted here is the ion density measured by the CIS/HIA instrument and the magnetic field is given by the FGM instrument. The data are normalised by the corresponding solar wind density and magnetic field shifted to 1 AU and obtained from the OMNI database^52^. The red lines are separatrix between three sectors following the work of Nguyen, *et al*.^6^, who defined those boundaries manually from similar plots made with data from the THEMIS, Double Star and Cluster missions. The small triangle at the bottom is associated with solar wind plasma, the larger triangle on the left is associated with regions located inside the magnetosphere and data located outside of these 2 triangles are associated with magnetosheath plasma. The percentage of data in each of these sectors for several regions and transition regions is given in Table 11. It shows that the GRMB regions are in good agreement with the sectorization introduced above^6,53^. 99.9% of Cluster-1 data in GRMB-PSH and 99.5% of Cluster-1 data in GRMB-PSTR are located in the pre-defined magnetosphere sector, 95.2% of Cluster-1 data in MSH are located in the pre-defined magnetosheath sector, and 89.3% of Cluster-1 data in SWF are located in the pre-defined solar wind sector. This shows that GRMB regions display different statistical properties and are well separated in the normalized density - magnetic field plane. This confirms that a direct use of the GRMB classification is well suited to make separate statistics on different regions. On the contrary, transition regions overlap between two sectors: Cluster-1 data in BSTR are located both in the solar wind (41.1%) and in the magnetosheath (50.6%) sectors and Cluster-1 data in the MPTR are located both in the magnetosheath (50.1%) and magnetospheric (50.6%) sectors. This is partly due to the properties of the transition regions that often consist of a mixture of plasma from the two adjacent regions but also to the definition of transition regions in the GRMB dataset: the transition regions can contain short time intervals of the adjacent regions. Indeed, the minimum time resolution of the GRMB dataset is 20 minutes. During periods when Cluster-1 skims the boundary between two regions, it can cross it several times and be alternatively located in the two adjacent regions for time periods shorter than 20 minutes. Such short time intervals will be identified as belonging to the transition region. Thus, if a user wants to select all the time intervals of one specific region, he must consider the time intervals identified as belonging to this region in the GRMB dataset and check the adjacent transition region. Note that the number of data in the transition regions is much lower than the number of data in the regions (see Table 11), so even if a manual check of the data in the transition region is necessary, it relates to a relatively small subset of data.

**Table 11:** Percentage of the GRMB dataset in 2007 for Cluster-1 located in each of the three sectors identified in Nguyen, *et al*.^6^.

GRMB label	Magnetosphere sector	Solar wind sector	Magnetosheath sector	Total number of data points
OUT/SWF	0.8%	89.3%	9.9%	1306512
OUT/BSTR	8.3%	41.1%	50.6%	212324
OUT/MSH	3.1%	1.7%	95.2%	1046025
IN/PSH	99.5%	0.2%	0.3%	1126855
IN/PSTR	99.9%	0.1%	0.0%	442827
IN/MPTR	47.6%	2.3%	50.1%	398954

Only regions where CIS/HIA can measure the plasma density are included.

#### Comparison between region properties: plasmasheet, plasmasheet transition region and lobes

**Fig. 16:**
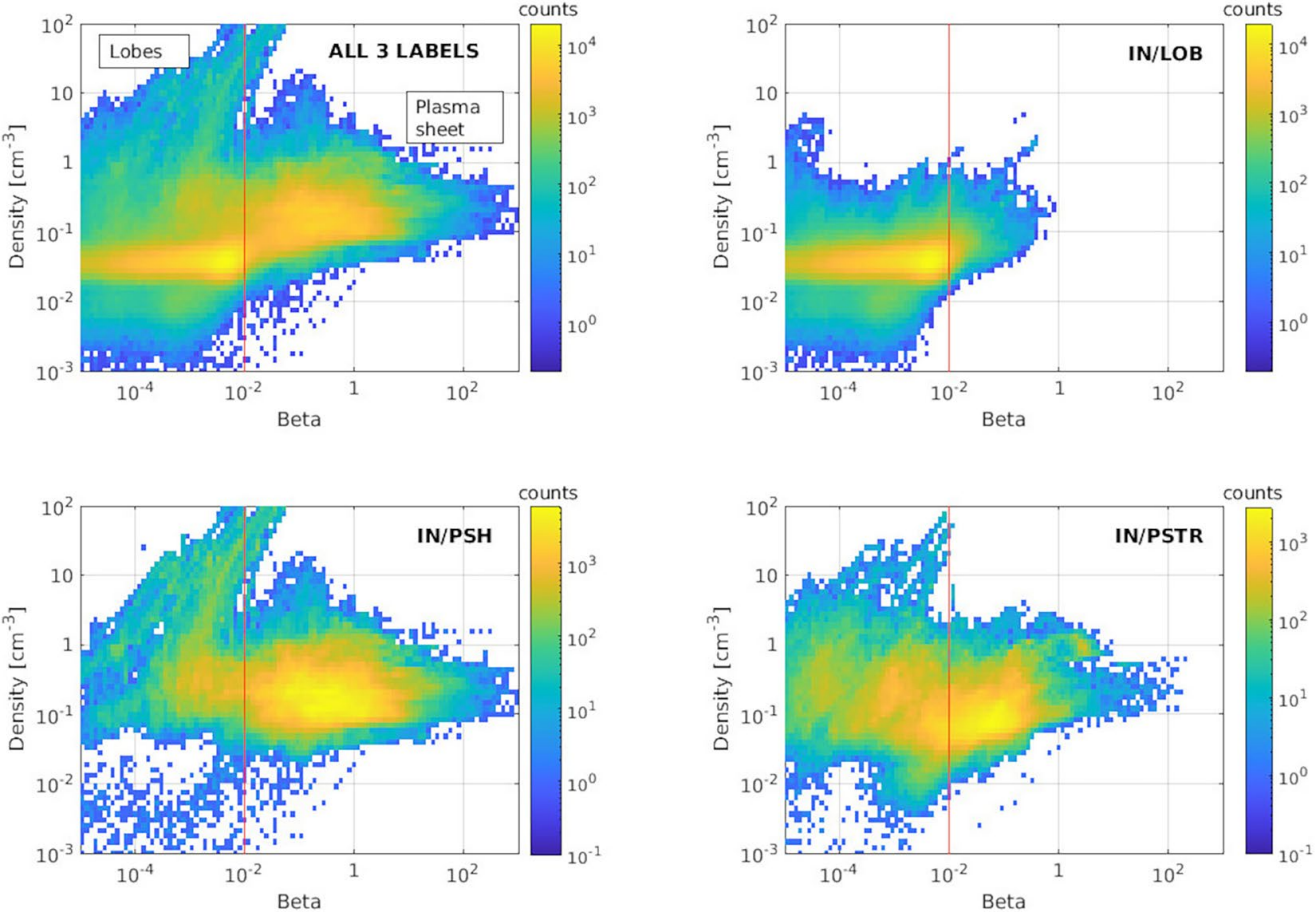
Two-dimensional histograms of the density measured by the CIS/HIA instrument as a function of the plasma *β* computed using CIS/HIA density measurements and FGM magnetic field measurements, onboard Cluster-1 in 2007. The regions are identified according to the GRMB dataset. Top left: for all regions and transition regions (IN/LOB, IN/PSTR, IN/PSH). Top right: for the lobe region (IN/LOB). Bottom left: for the plasmasheet (IN/PSH). Bottom right: for the plasmasheet transition region (IN/PSTR). The data have been obtained from the Cluster Science Archive (CSA). The red vertical line represents a visually determined separatrix corresponding to a plasma *β* of 10^−2^. It uses this simple criteria on the plasma *β* to separate the lobe region (on the left) from the plasmasheet (on the right).

The second example of application concerns regions located inside the magnetosphere: the lobes (LOB), the plasmasheet transition region (PSTR) and the plasmasheet (PSH). Figure 16 shows the distribution of Cluster-1 data in 2007 for those three regions respectively in a plasma *β* - density plane. The density is obtained from the CIS/HIA instrument and the plasma *β* is computed using the plasma temperature from CIS/HIA and the magnetic field from the FGM instrument. In this second example, the red lines correspond again to a visually determined separatrix, here between the plasmasheet sector on the right side of the red line and the lobe sector in Fig. 16). This shows that the GRMB regions have distinct statistical properties, while the transition regions, here the plasmasheet transition region, overlap between the plasmasheet and the lobe sectors (see Table 12). A large majority of the data in the lobe region lies in the lobe sector (95% in the plasma *β* - density plane and 98.6% in the density - temperature plane) and a large majority of the plasmasheet region in the plasmasheet sector (89% in the plasma *β* - density plane). In this example too, the transition region has properties that overlap with the properties of the two adjacent regions. Data in the plasmasheet transition region are located both in the plasmasheet and lobe sectors (41.4% and 58.6% in the plasma *β* - density plane). This is due to the intrinsic properties of the plasmasheet transition region, which may have intermediate density, plasma *β* and temperature values between the lobes and plasmasheet but also to the presence of shorter than 20 minutes time intervals of lobes or plasmasheet in the GRMB plasmasheet transition region when Cluster-1 skims the boundary between these two regions. This highlights a difference between the approach of our classification, i.e. including time short intervals of the adjacent regions in the transition region, compared to other classifications. For instance, in the ECLAT database^48^, the distinction between the lobes, the outer plasmasheet and the boundary region between them is based on a condition on the plasma *β*. In the ECLAT database, the plasmasheet, lobes and boundary regions have by definition different properties that cannot overlap in the plasma *β* - density plane. With such a sharp limit between regions, it cannot be excluded that some plasmasheet boundary layer intervals (PSBL, corresponding to the PSTR in the GRMB dataset and to the Boundary Region or BR in the ECLAT database) are included in the lobe or plasmasheet region. However, such automated approach provides a much shorter time resolution than in the GRMB dataset where regions are identified visually. Similar to the conclusions of the previous example, if a user wants to select all the time intervals of one specific region, for instance the lobes region, she/he must consider the time intervals identified as belonging to LOB in the GRMB dataset but must also check the adjacent transition region, i.e. the PSTR region. If a user wants to analyze the statistical properties of one region, for instance the lobes, he/she may not need to consider the plasmasheet transition region as only a limited proportion of the time periods when Cluster is located in the lobes is included in the PSTR region.

**Table 12:** Percentage of the GRMB dataset located in each of the two sectors identified in Fig. 16.

GRMB label	Plasmasheet sector	Lobe sector	Total number of data points
IN/LOB	5.0%	95.0%	1 311 196
IN/PSH	89.0%	11.0%	1 126 856
IN/PSTR	58.6%	41.4%	442 828

## Usage Notes

### Continuous coverage

A few rules are necessary to get a homogeneous dataset. The GRMB dataset is continuous by construction: for each time during the Cluster mission, there is one label per spacecraft. The operator selects a label at the item (region or transition region) start time. An item ends at the start time of the next label. It is thus mandatory to select a label at every change of region (no data, unknown, inside, outside, defined region or defined boundary).

### Time resolution

The default time resolution is 20 minutes. That was found to be the optimum duration to produce the dataset within a reasonable time frame. If there is a succession of several regions shorter than 20 minutes, only the most important is selected, possibly with the flag *complex*. PLS and MP can be occasionally shorter than 20 minutes. Transition regions (PPTR, MP, MPTR, POL, BSTR) can be shorter than 20 minutes when sharp boundaries are observed. We expect that PSTR will last longer than 20 minutes.

### Regions overlap

Transition regions may have properties from the neighboring regions in order to include the full boundary. For example:

- LOB, POL and PSH properties can be found in PSTR.
- MSH, PSH, PSTR, and LOB can be found in MPTR, MP, and POL.
- MSH and SWF can be found in BSTR.

As a result LOB and PSH should be more homogeneous than PSTR; MSH and SWF more homogeneous than BSTR.

It is known that some regions can overlap in the magnetosphere. We defined two priorities:

- PSH and PSTR can overlap with POL: priority is given to POL identification given the Cluster polar orbit.
- PSH, PSTR and POL can overlap PPTR and PLS: priority is given to PPTR and PLS identification. Along the Cluster orbit energetic plasma populations are often seen in the plasmapause and the plasmasphere regions. When one wants to include more intervals with energetic particle regions it is worth adding PLS and PPTR items to PSH (and PSTR).

If a user selects one of the PLS, PSH, LOB, MSH or SWF labels he/she will get a dataset for this region of fairly good quality.

The labels PPTR-PLS, MP-MPTR, POL and BSTR are useful to study respectively the plasmapause, the magnetopause, the polar regions and the bow shock. It is recommended to apply some criteria to obtain a fully homogeneous sub-dataset.

For example, to perform extensive studies it is good to include the following labels (and possibly apply criteria afterwards):

- LOB: Add PSTR (and possibly POL).
- POL: Add PSTR, PLS, LOB and MPTR.
- PSH: Add PSTR, LOB, PPTR, MPTR, POL and PLS.
- PSTR: Add LOB, PSH, POL.
- MP, MPTR: Add POL.
- MSH: Add BSTR, MP, MPTR, POL.
- SWF: Add BSTR.

For studying a region not listed in our GRMB labels, the user can combine the labels matching some of the properties of the target. Selecting the IN or the OUT labels is the first step of this.

### Forbidden consecutive items

Before validation, a check is made to catch some possibly incorrect labels. The operator has to modify the selection according to the following rules:

- There is always an MP, MPTR or POL label between an IN and an OUT label.
- There is always a BSTR (or OUT/UKN, or UKN) label between MSH and SWF labels.
- There is always another IN label between PLS, PPTR and an IN-OUT region (POL can occur but there is a need to double-check).

These rules make certain that for a given orbit where Cluster enters the SWF region, there will be at least two BSTR, at least two magnetopause-related items (MP-MPTR-POL), and at least one IN label (and at least three if there is a PPTR).

### Limitation and caveats

The dataset does not resolve regions where the spacecraft spends only a brief time interval, typically less than 20 minutes. This is why properties can be mixed within a single entry. When there is a succession of short LOB and PSTR properties, it should be listed as PSTR. But if this succession appears inside a long LOB region, it can remain labelled as LOB (in that case the tag complex should be used).

This is a “by-eye”classification performed by different operators. Even if the instructions are the same for everyone, there are some differences of interpretations for different operators, but also different interpretations over time for a single operator. To limit this bias, instruction is given to take large transition regions. Regions are thus “cleaner” than transition regions which may include short time periods when Cluster is located in the adjacent region.

The most difficult identification is the distinction between PSTR, LOB (with *crossing*_*complexity* activated, including low-energy ionospheric outflows) and POL. These geospace regions can be found at the same location and have a similar particle energy distribution. Identification is especially difficult when no ion data are available. POL is usually located in the [8-16] MLT range. The PSTR ion population is supposed to be more isotropic than POL and LOB populations. The LOB ion population is supposed to be less energetic than POL and PSTR populations. In case of doubt, IN/UKN is the recommended solution. Many IN regions require particle measurements for their identification, and especially ion measurements. It is much more difficult to identify a POL when no ion measurements are available; disentangling PSH, PSTR and LOB as well. When no particle measurements are available, IN/UKN entries are more numerous. In that respect the identification of the OUT regions is more reliable than the IN regions.

There might be a confusion between POL (mid-altitude) and PPTR due to the density increase seen in WHISPER in some cusps crossings. In that case, the particle energy, the MLT, and the distance to Earth can help making a choice. Once again, the complex tag is used to mark the presence of properties matching several items and the IN/UKN is used when it does not clearly match any item.

The identification is based on the pre-generated CSA plots. The shortest duration of those plots is 1 hour. Some short duration boundary crossings can thus be missed. It is not possible for an operator to visualize all the available data for each region selection. For a given parameter the tool shows the product that contains the most data. In case of doubt the operator can check any panel. A posteriori, the user can check which panels were displayed at the selection time. The panels considered for the label selection are reflected in the quality flag.

## Data Availability

The GRMB dataset relies on a visual identification of the boundaries based on pre-generated plot available at the CSA. There is no specific code to be made available.
